# Spatiotemporal Fusion for Stock Prediction via Hypergraph Attention Gated Recurrent Units

**DOI:** 10.3390/e28050517

**Published:** 2026-05-03

**Authors:** Xinmei Cao, Chonghui Qian, Hengjun Huang

**Affiliations:** 1 School of Statistics and Data Science, Lanzhou University of Finance and Economics, Lanzhou 730020, China; caoxm@lzufe.edu.cn; 2 Key Laboratory of Digital Economy and Social Computing Science of Gansu, Lanzhou 730020, China

**Keywords:** stock prediction, recurrent spatiotemporal fusion, hypergraph attention, gated recurrent unit, tucker decomposition, higher-order stock dependence

## Abstract

Stock prediction requires the joint modeling of temporal dynamics and cross-stock dependence. Existing graph-based and hypergraph-based forecasting methods often process spatial relation modeling and temporal evolution in separate stages, which may weaken the interaction between relational information and recurrent state updating. This study proposes a Recurrent Spatiotemporal Hypergraph Attention Gated Recurrent Unit model for stock forecasting, in which hypergraph-based higher order dependence and temporal dynamics are integrated within each recurrent update. The hypergraph is constructed offline from heterogeneous financial features through Tucker decomposition, similarity estimation, and Top-K sparsification, and is then used as a structured relational prior during forecasting. Experiments on CSI 300 constituent stocks from January 2014 to October 2024 show that RST-HGA-GRU achieves the best overall performance across multiple evaluation metrics and forecasting horizons from 1 to 6 days. Ablation, sensitivity, back testing, and multi-horizon Diebold–Mariano tests further support the effectiveness and robustness of the proposed framework. These results demonstrate that recurrent spatiotemporal fusion with hypergraph-based higher-order relation modeling is effective for stock price forecasting.

## 1. Introduction

Stock price forecasting remains a fundamental yet challenging problem in financial research because it directly supports portfolio allocation, risk management, and market monitoring. In real markets, stock prices are influenced by multiple factors, including historical trading dynamics, firm fundamentals, sectoral interactions, and broader market conditions. The joint effect of these heterogeneous factors leads to strong nonlinearity, noise, and uncertainty in financial time series, which makes accurate forecasting difficult.

Traditional forecasting methods, such as Autoregressive Integrated Moving Average (ARIMA) and Long Short-Term Memory (LSTM) [1,2], mainly focus on temporal dependence within individual price sequences. These models are effective in capturing local sequential patterns, but they often provide only a partial view of market behavior. In practice, stock movements are rarely independent. Firms may co-move because they belong to the same industry, share similar financial characteristics, or respond jointly to common market and policy shocks. Therefore, modeling only temporal dynamics may overlook important cross-stock dependence that is relevant to forecasting performance.

Financial data are inherently multi-source and relational. Market variables such as prices, trading volume, return volatility, and beta describe dynamic trading behavior, whereas fundamental indicators such as earnings per share, net assets per share, and growth-related measures reflect firm-level financial conditions [3,4]. These heterogeneous features contain both temporal information and structural information. Temporal information characterizes the evolution of each stock over time, while structural information reflects similarity, interaction, and dependence among stocks. A forecasting framework that integrates both aspects is therefore more appropriate for complex financial markets than a model based on time series signals alone.

Recent studies have introduced graph-based and hypergraph-based spatiotemporal learning into stock forecasting [5,6,7,8,9,10,11,12,13,14]. These studies show that relational structure can improve predictive performance by modeling cross-stock dependence beyond pure temporal signals. Representative hypergraph-based models, including the Dynamic Hypergraph Spatiotemporal Network (DHSTN) [11], the Attention-based Adaptive Spatiotemporal Hypergraph Convolutional Network (AASTHGCN) [12], the Market-wide Dynamic Hypergraph Attention Network (MDHAN) [13], and the Hypergraph Tri-Attention Network (HGTAN) [14], further demonstrate the value of higher order relation modeling in practical forecasting tasks. In many existing frameworks, relational modeling and temporal updating are implemented as functionally separate stages. A graph or hypergraph module is commonly used to encode spatial dependence, and the resulting representation is then combined with a temporal predictor. Under such a formulation, higher order dependence contributes to forecasting through relation-aware features, while recurrent state evolution is mainly governed by the temporal module.

A related issue concerns relation construction. Existing stock relation modeling often relies on prior knowledge, pairwise similarity, or a single data source, which may not adequately reflect the heterogeneous and higher order dependence embedded in financial markets [15,16,17]. Relation structures derived directly from raw features can also be affected by redundancy, scale heterogeneity, and short-term noise, which may reduce their effectiveness as relational priors for downstream forecasting.

Motivated by these observations, this study develops a recurrent spatiotemporal forecasting framework in which higher order stock dependence is incorporated into the recurrent updating process. Specifically, a stock hypergraph is constructed offline from heterogeneous financial features through Tucker decomposition, similarity estimation, and Top-K sparsification, and is then reused as a structured relational prior during forecasting. On this basis, hypergraph-based relational aggregation is integrated into the GRU state transition, allowing cross-stock dependence and temporal dynamics to interact within each recurrent update.

The main contributions of this study are as follows:

(1) A recurrent spatiotemporal forecasting framework is developed by integrating hypergraph-based relational aggregation into the GRU state transition. This formulation enables higher order cross-stock dependence to participate in hidden state updating throughout the forecasting process.
(2) An offline stock relation construction scheme is designed from heterogeneous financial features through Tucker decomposition, similarity estimation, and Top-K sparsification. The resulting hypergraph provides a structured representation of pairwise affinity and higher order group dependence among stocks.
(3) The proposed formulation provides a unified recurrent setting for temporal dynamics and higher order relational aggregation, which strengthens the interaction between cross-stock dependence and hidden state evolution.
(4) Extensive experiments on CSI 300 constituent stocks across forecasting horizons from 1 to 6 days are conducted, together with ablation analysis, sensitivity analysis, back testing analysis and multi-horizon Diebold–Mariano tests, to examine the effectiveness and robustness of the proposed framework.

The remainder of this paper is organized as follows. Section 2 reviews related work on forecasting models and their applications to stock price prediction. Section 3 outlines the study methodology, including an overview of the proposed approach, financial association network construction, and the fusion modeling algorithm. Section 4 presents the experimental design, benchmark settings, results, and discussion. Finally, Section 5 concludes the study by summarizing the key findings and contributions to stock price prediction, and discusses the study’s limitations.

## 2. Related Work

This section provides an overview of temporal-based and spatiotemporal-based stock price prediction models, along with methods for constructing spatial correlation networks in stock price forecasting.

### 2.1. Temporal-Based Models

Accurate stock price prediction relies on a variety of temporal factors, including market trends, economic cycles, and corporate earnings reports. Temporal-based models, such as ARIMA, Support Vector Regression (SVR), Random Forest Regression (RFR), and eXtreme Gradient Boosting (XGBoost), have been widely used to capture periodic patterns and temporal dependencies, thereby enabling effective forecasting [2,18,19,20,21,22,23]. For example, ARIMA has demonstrated competitive performance in short-term stock prediction tasks [2]. To better capture nonlinear relationships and long-term dependencies, machine learning approaches have been increasingly adopted [18,19,20,21]. XGBoost has been utilized for feature selection in high-dimensional time-series data, effectively identifying relevant features and eliminating redundancy [20]. Additionally, hybrid models, such as SVR combined with the Equilibrium Optimizer, have been proposed for predicting closing prices on markets like the Egyptian Exchange. While machine learning models offer greater flexibility in modeling nonlinear patterns, they often require time-consuming manual feature engineering, complex hyperparameter tuning, and may face scalability challenges when applied to large-scale datasets.

In recent years, deep learning has been widely adopted in stock price prediction due to its strong capability to extract discriminative features and model complex temporal dependencies [24]. Various deep learning architectures have been applied to this task, including Temporal Convolutional Networks (TCNs) [25], LSTM networks [26,27,28] and GRU networks [29,30,31]. As an illustration, an enhanced TCN model incorporating a channel attention mechanism was proposed to improve prediction performance [25]. Additionally, LSTM, Recurrent Neural Networks (RNNs), and GRU models have been extensively implemented and fine-tuned for stock forecasting tasks [32]. While traditional machine learning methods are relatively simple, they often lack the accuracy and robustness required for complex financial prediction. In contrast, deep learning models demonstrate superior nonlinear modeling capabilities and higher predictive accuracy, making them more suitable for stock price forecasting.

### 2.2. Spatiotemporal-Based Models

The application of deep learning has significantly advanced multi-source feature fusion in stock price prediction, particularly in integrating spatial correlations with historical trading data to enhance forecasting accuracy. Spatiotemporal models, commonly implemented through GNNs and Hypergraph Neural Networks (HGNNs), jointly capture spatial dependencies among stocks and temporal patterns in price movements, thereby improving prediction robustness [9,10,11]. These models typically consist of a spatial module to extract inter-stock relationships and a temporal module to model historical dynamics. Several spatiotemporal models have been developed for stock prediction, including the DHSTN [11] and the AASTHGCN [12]. For instance, DHSTN employs GRU to learn temporal embeddings and a dynamic hypergraph to model stock relationships, achieving improvements of at least 4.99% in F1-score and 47.9% in Sharpe ratio on the CSI 300 and NASDAQ-100 datasets. Similarly, the MDHAN integrates an attention-based market-aware GRU and hypergraph neural network to capture temporal and spatial features, respectively, and demonstrates superior performance across four real-world datasets from China and the U.S. [13]. In addition, the HGTAN leverages GRU for temporal encoding and hypergraph convolution with hierarchical attention for capturing group-wise spatial dependencies, achieving strong performance in practical scenarios [14]. Despite their effectiveness, these models typically adopt a sequential (tandem) approach to feature fusion, extracting temporal and spatial features separately, which hinders embedded spatiotemporal fusion. Moreover, while GNNs can model spatial relationships, they often struggle to capture high-order interactions among stocks, limiting their ability to represent global market structures accurately.

### 2.3. Stock Spatial Correlation Network Construction

In recent years, stock correlation networks have gained increasing attention in stock price prediction research for their ability to capture co-movement effects and transmission mechanisms of price fluctuations. These networks typically represent stocks as nodes and their interrelationships as edges, and are commonly constructed using three approaches: prior knowledge-based, knowledge graph-based, and similarity-based methods. Prior knowledge-based methods build networks using explicit relationships such as industry affiliation [33], supply chain links [34], or cross-shareholding structures [35], offering intuitive interpretations but relying heavily on high-quality domain knowledge and often failing to capture latent interactions. Knowledge graph-based methods extract inter-stock relationships by integrating structured data sources, such as financial reports and news, using techniques like text mining and event extraction [16,36,37,38,39]. Although semantically rich, these methods are complex and data-intensive. Similarity-based methods, such as those based on Pearson correlation or dynamic time warping [6,7,40], identify latent connections by measuring similarities in price sequences, yet they are sensitive to noise and lack interpretability in economic contexts. Despite their contributions, existing approaches typically emphasize single-type relationships while overlooking complex and latent interactions, including investor sentiment and multi-source data correlations; consequently, their robustness and expressiveness are limited.

Overall, existing studies have demonstrated the usefulness of graph-based and hypergraph-based relation modeling for stock forecasting. At the same time, there remains room to further strengthen the interaction between relational dependence and recurrent temporal updating, and to improve the stability of relation construction under heterogeneous financial features. These considerations motivate the framework proposed in this study, which combines tensor-based hypergraph construction with recurrent spatiotemporal fusion for stock forecasting.

## 3. Materials and Methods

### 3.1. Data Description

This study focuses on the constituent stocks of the China’s CSI 300 Index and selects 100 stocks that remained continuously included in the index from 1 January 2014 to 31 October 2024, as shown in Table 1. This sampling strategy is adopted to reduce data discontinuities caused by constituent changes and to provide a stable stock universe for longitudinal forecasting analysis. Since the hypergraph construction and recurrent forecasting framework both rely on consistent panel structure over time, a continuously included stock set helps avoid repeated interruptions in relation to construction and sample generation. At the same time, this choice may limit sample representativeness because it places greater emphasis on relatively stable, large-cap, and highly liquid constituent stocks while excluding stocks with shorter inclusion periods or stronger membership turnover. Therefore, the empirical findings of this study should be interpreted primarily for a stable CSI 300 large-cap setting rather than as a complete characterization of the full evolving constituent universe. Stocks lacking sufficient historical observations, such as stock 000333, which was listed in September 2013, were excluded to ensure the completeness of the factor-based analysis.

The final dataset covers 2633 trading days and contains 100 stocks with 28 feature indicators, resulting in a tensor of dimensions 2633 × 100 × 28. To construct the stock hypergraph incidence network, this study incorporates a comprehensive set of variables from four categories: historical market data, stock-specific risk factors, financial style indicators, and valuation metrics. Historical market data include adjusted closing price, trading volume, price change, and turnover rate. Stock-specific risk factors include return rate, return volatility, Beta, correlation coefficient, and unsystematic risk. Financial style indicators include earnings per share, net asset value per share, revenue per share, operating cash flow per share, stock value score, earnings growth rate, net asset growth rate, revenue growth rate, operating cash flow growth rate, stock growth score, and a value-growth hybrid score. Valuation metrics include price-to-earnings ratio, price-to-book ratio, price-to-cash-flow ratio, price-to-sales ratio, enterprise value, enterprise value multiple, dividend yield, and A-share market capitalization. These variables capture both dynamic behavior and fundamental attributes of individual stocks and thus provide a multi-dimensional basis for constructing a structurally informative hypergraph network. Their inclusion helps characterize long-term stock interactions from complementary perspectives and supports the modeling of complex financial correlations. The dataset is obtained from the CSMAR database (https://data.csmar.com/).

To reduce the impact of scale differences and improve model convergence, the dataset is normalized using Min-Max scaling as defined in Equation (1),

(1) $$x^{\prime }=\frac{x-\min(x)}{\max(x)-\min(x)}$$

where *x* and $x^{\prime }$ are the feature values before and after normalization, respectively.

To avoid information leakage, all preprocessing steps were conducted in chronological order. For each experimental split, normalization was fitted using the training period only and then applied to the corresponding validation period. Likewise, the spatial structure used by graph-based and hypergraph-based models was constructed from the training window only and then reused in the subsequent validation phase. No statistics from future observations were used during preprocessing, hypergraph construction, or model training. This setting ensures that all reported results were obtained under a strictly forward-looking forecasting protocol.

### 3.2. Overview

**Figure 1:**
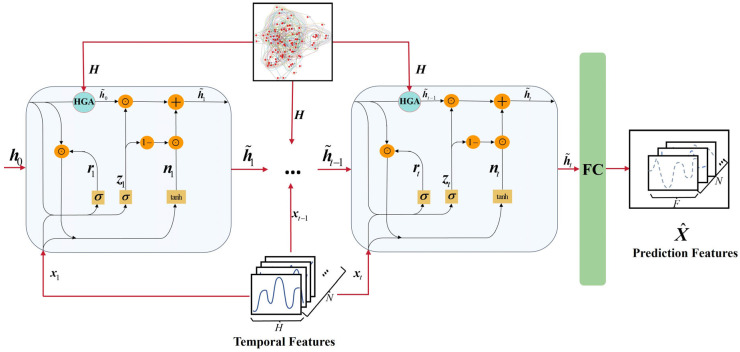
The framework of RST-HGA-GRU model.

This section outlines the implementation of the RST-HGA-GRU model. As illustrated in Figure 1, the model performs recurrent forecasting by combining temporal hidden-state evolution with hypergraph-based relational aggregation. At each time step, the GRU processes the current stock features and the previous hidden state, while the HGA module aggregates higher-order stock dependence from the hypergraph structure. These two information streams are then fused within the recurrent update, and the final hidden representation is passed to a fully connected layer for prediction. This tightly coupled design enables higher-order stock dependence to participate directly in hidden-state evolution, thereby improving recurrent spatiotemporal forecasting.

The stock price prediction task can be formulated as a process in which a parameterized model is trained using the historical closing prices of *N* stocks (*x_t_*_−*H*+1_, …, *x_t_*_−1_, *x_t_*) and a hypergraph spatial incidence matrix (***H***) to predict the future stock close prices (${\hat{x}}_{t+1}$, …, ${\hat{x}}_{t+F}$), as shown in Equation (2),

(2) $$\left[{\hat{x}}_{t+1},\ldots ,{\hat{x}}_{t+F}\right]=f_{\theta }\left((H);\left(x_{t-H+1},\ldots ,x_{t-1},x_{t}\right)\right)$$

where *F* represents the forecast horizon, *t* denotes the current time step, and *H* represents the history length used in the model.

The model leverages the HGA module to effectively capture higher-order interactions between the nodes in the stock network. By constructing the hypergraph and utilizing a dynamic attention mechanism, it reflects the complex relationships between stocks, thereby facilitating the capture of higher-order interactions. Meanwhile, the GRU component addresses the long-term dependencies in the sequential data through its gating mechanism, allowing efficient extraction of temporal features. The embedded design of the HGA module and the GRU enables the model to dynamically fuse spatial features with temporal features, overcoming the limitations of traditional methods where spatiotemporal features are treated separately. This framework, combining the advantages of hypergraph networks and recurrent neural networks, allows the model to better capture spatiotemporal features, thereby achieving improved prediction accuracy in stock price forecasting tasks.

### 3.3. Construction of Stock Hypergraphs Based on Tensor Decomposition

To effectively capture the complex interactions among time, stocks, and various financial features, the original data is represented as a three-dimensional tensor $X\in {\mathbb{R}}^{T\times N\times F}$, where *T*, *N* and *F* denote the temporal, stock, and feature dimensions, respectively. Compared with direct matrix-based processing, this tensor representation preserves the intrinsic multi-mode structure of the data and is therefore more suitable for describing the complex dependence patterns embedded in multi-source financial variables. In particular, the stock relation construction stage should not rely solely on raw pairwise similarity computed from the original high-dimensional features, because such similarities can be strongly affected by scale heterogeneity, redundant dimensions, and short-term noise. For this reason, we first derive a compact latent representation before constructing the stock hypergraph.

To mitigate noise and reduce dimensionality while preserving cross mode interactions, Tucker decomposition is applied to the normalized input tensor ***X***, as defined in Equation (3):

(3) $$X\approx G\times_{1}A\times_{2}B\times_{3}C$$

The decomposition yields a core tensor $G\in {\mathbb{R}}^{R_{t}\times R_{n}\times R_{f}}$ and three factor matrices **A**, **B** and **C**, which jointly capture the latent interactions among the temporal, stock, and feature modes. The reduced ranks satisfy *R_t_* < *T*, *R_n_* < *N*, and *R_f_* < *F*. Tucker decomposition is adopted here for two reasons. First, it preserves the three-way structure of the original tensor and jointly models the interactions among temporal evolution, stock identities, and heterogeneous features, which is preferable to flattening the data into a matrix before similarity estimation. Second, it provides a low rank latent representation that suppresses local noise and redundant variation, making the subsequent stock relation construction more stable and more informative. Before decomposition, all input variables are normalized to ensure comparable scales and to reduce the dominance of variables with large numerical magnitudes. The Tucker ranks (*R_t_*, *R_n_*, *R_f_*) are selected by principal component analysis (PCA) along the corresponding tensor modes. This PCA-based strategy is used as a data-driven compromise between information preservation and representation compactness. In the context of hypergraph construction, overly small ranks may discard informative cross-stock structure, whereas overly large ranks may reintroduce noise and weaken the stability of the resulting similarity matrix. Therefore, PCA-based rank selection provides a practical way to retain the dominant variance of each mode while avoiding unnecessarily large latent dimensions.

(4) $$U_{N}=G\times_{2}B$$

(5) $$U_{N,\mathrm{aggregated}}=\mathrm{mean}(U_{N},\mathrm{axis}=0)$$

(6) $$\mathrm{similarity}(i,j)=\frac{u_{i}^{T}u_{j}}{\left\|u_{i}\|\|u_{j}\right\|}$$

(7) $$E_{i}=\left\{j|j\in S_{i}^{K}\right\}$$

After decomposition, the stock feature matrix is reconstructed from the stock factor matrix **B** and the core tensor **G** according to Equation (4). This reconstructed representation preserves the main latent commonalities and differences among stocks in a lower dimensional form. Subsequently, Equation (5) is used to aggregate the reconstructed features over time. The purpose of this mean aggregation step is not to replace temporal modeling in the forecasting network, but to obtain a more stable stock level structural descriptor for relation construction. By smoothing short term fluctuations and transient noise, this step helps prevent the hypergraph from being dominated by highly local and irregular variations that are less useful for identifying persistent stock associations.

Based on the aggregated latent stock representation $U_{N,\mathrm{aggregated}}$, pairwise stock similarities are then computed using cosine similarity, as defined in Equation (6), which yields the similarity matrix $S\in {\mathbb{R}}^{N\times N}$. Cosine similarity is adopted because the relation construction stage focuses primarily on structural alignment in the latent space rather than on absolute feature magnitude. After normalization and low rank reconstruction, the direction of the stock representation vector is more informative than its raw scale for identifying stocks with similar latent profiles. In this sense, cosine similarity is well suited for measuring relative proximity among stocks in the denoised latent representation space.

To reduce computational redundancy and retain only the most informative local relations, a Top-K sparsification strategy is applied to the similarity matrix. Specifically, for each stock *i*, the *K* most similar stocks are selected to form a hyperedge, as described in Equation (7). This design is motivated by two considerations. On the one hand, a fully connected relation structure may dilute informative local dependence and introduce excessive noise into subsequent spatial learning. On the other hand, Top-K sparsification preserves the strongest neighborhood affinities for each stock and yields a more interpretable and computationally efficient hypergraph structure. The resulting hypergraph is denoted as H=(V,ε), where V represents the set of all stock nodes and $\varepsilon =\left\{E_{1},E_{2},\ldots ,E_{n}\right\}$ $\varepsilon =\left\{E_{1},E_{2},\ldots ,E_{n}\right\}$ denotes the set of hyperedges. Because each hyperedge can connect multiple stocks simultaneously, the constructed hypergraph is able to represent not only pairwise associations but also higher order group interactions and collective behavior in the stock market.

Overall, the hypergraph construction procedure is designed to balance structural expressiveness, robustness, and computational tractability. Tucker decomposition is used to derive a denoised latent representation from multi-source financial data, PCA-based rank selection controls representation complexity, mean aggregation reduces transient fluctuations during relation construction, and Top-K sparsification prevents the hypergraph from becoming overly dense. In this way, the resulting incidence structure serves as a stable relational prior for the subsequent hypergraph-based spatial modeling in Section 3.4. The robustness of this construction procedure with respect to the Top-K parameter and the PCA threshold for Tucker rank selection is further examined in Section 4.4.3.

**Figure 2:**
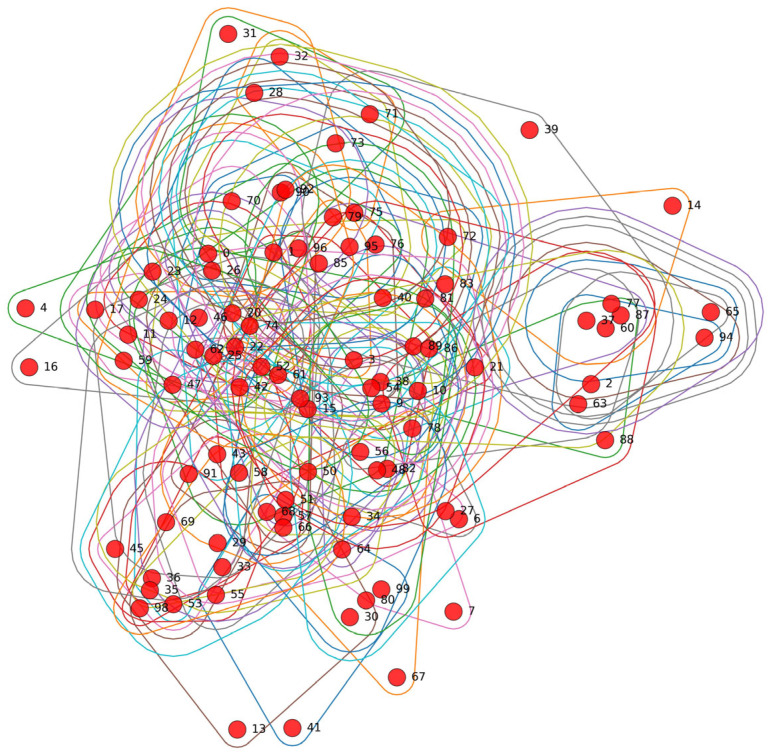
Visualization of stock hypergraph network structure.

Figure 2 illustrates the stock market network structure derived from the hypergraph incidence matrix using the Top-K method with *K* = 5. In this representation, each node corresponds to a stock, while each hyperedge reflects a higher-order relation among multiple stocks. The network exhibits a relatively dense connectivity pattern, suggesting substantial interactions and structural dependence across the market. Some nodes occupy more central positions within the network, indicating that they may play a stronger role in reflecting shared market conditions or common information within the constructed dependence structure. These connectivity patterns provide an intuitive illustration of the higher-order stock dependence captured by the proposed hypergraph construction method.

From an economic perspective, the constructed hypergraph should be interpreted as a structural dependence prior rather than as a direct causal network. Because the hypergraph is built from both trading-related and fundamental features, the learned hyperedges may reflect shared industry membership, common exposure to market-wide or macroeconomic shocks, similarity in valuation and growth characteristics, and co-movement induced by common risk transmission channels. In this sense, dense local connectivity suggests that groups of stocks may respond in a similar way to underlying market conditions, while relatively central nodes may act as representative carriers of common information or shared shocks within the market structure. This interpretation helps explain why hypergraph-based aggregation is useful for forecasting, as it allows the model to exploit not only pairwise similarity but also economically meaningful group-level dependence among stocks.

### 3.4. RST-HGA-GRU Model

#### 3.4.1. Spatial Features Modeling

Spatial relation modeling is important for stock forecasting because it captures dependence among stocks beyond individual temporal trajectories. Compared with pairwise graph models such as GCN and GAT, hypergraph attention aggregation is more suitable for the present task because it can represent higher-order interactions among multiple stocks within the same hyperedge. Therefore, this study adopts the HGA module to aggregate higher-order stock dependence from the hypergraph constructed in Section 3.3.

This study uses the hypergraph incidence matrix constructed in Section 3.3 for spatial feature modeling. The HGA module is adopted to aggregate stock relation information in a higher-order form, allowing the model to move beyond uniform pairwise aggregation and better capture complex stock interactions. The implementation process is detailed as follows:

(8) $$\alpha_{ie}=LR(a^{T}[Wh_{i};Wh_{e}])$$

(9) $${\tilde{\alpha }}_{ie}=\frac{\exp(\alpha_{ie})}{\sum_{e^{\prime }\in {\bar{E}}_{i}}\exp(\alpha_{ie^{\prime }})}$$

(10) $${\tilde{h}}_{i}=HGA(h_{e})=\sum_{e\in {\bar{E}}_{i}}{\tilde{\alpha }}_{ie}Wh_{e}$$

where LR is the Leaky ReLU activation function, $a\in {\mathbb{R}}^{2d^{\prime }}$ and **W** are the learnable parameters, $\left[\cdot ;\cdot \right]$ means concatenation, ***h****_i_* is the historical hidden feature of stock *i*, $h_{e}=\frac{1}{\left|e\right|}\sum_{j\in e}x_{j}$ is the historical hidden feature of the hyperedge, ${\bar{E}}_{i}$ is the set of all hyper edges of stock *i*. Equation (8) is used to calculate the correlation between stock *i* and hyperedge *e*, a larger value indicates a stronger correlation between stock *i* and hyperedge *e*. Equation (9) is used to normalize the correlation between stock and hyperedge using Softmax function to get the normalized correlation ${\tilde{\alpha }}_{ie}$. Then, Equation (10) is used to ${\tilde{h}}_{i}$. The hypergraph attention module effectively captures the spatial correlation features of the stock data and focuses on the correlation between stocks and hyper edges through the attention mechanism, resulting in an effective spatial feature modeling capability.

#### 3.4.2. Temporal Features Modeling

Temporal dependence is modeled by a GRU because it provides an efficient gated recurrent mechanism for capturing sequential information in stock price series. Compared with more complex recurrent architectures, GRU offers a compact structure and competitive forecasting performance, which makes it suitable for the present framework. The recurrent update is defined by Equations (11)–(14):

(11) $$r_{t}=\sigma \left(W_{ir}x_{t}+b_{ir}+W_{hr}h_{t-1}+b_{hr}\right)$$

(12) $$z_{t}=\sigma \left(W_{iz}x_{t}+b_{iz}+W_{hz}h_{t-1}+b_{hz}\right)$$

(13) $$n_{t}=\tanh\left(W_{in}x_{t}+b_{in}+r_{t}⊙(W_{hn}h_{t-1}+b_{hn})\right)$$

(14) $$h_{t}=\left(1-z_{t}\right)⊙n_{t}+z_{t}⊙h_{t-1}$$

where ⊙ denotes the Hadamard product, σ denotes the sigmoid activation, and tanh denotes the hyperbolic tangent activation. **W***_ir_*, **W***_hr_*, **W***_iz_*, **W***_hz_*, **W***_in_* and **W***_hn_* are learnable parameter matrices, **b***_ir_*, **b***_hr_*, **b***_iz_*, **b***_hz_*, **b***_in_* and **b***_hn_* are learnable bias vectors. ***r****_t_* and ***z****_t_* denote the reset gate and update gate, respectively, while ***n****_t_* denotes the candidate hidden state. Equation (14) updates the current hidden state by combining retained historical information with newly generated information from the current input.

#### 3.4.3. Features Fusion Modeling

Stock spatiotemporal data are highly dynamic and complex, requiring the model to capture informative patterns from multiple feature dimensions in a timely and accurate manner. Such data usually contain heterogeneous information across temporal, cross-sectional, and structural dimensions. Therefore, effective spatiotemporal fusion is essential for improving the accuracy and reliability of stock forecasting.

In this study, spatiotemporal feature fusion refers to the integration of temporal dynamics and hypergraph-based spatial dependencies within each recurrent update. Specifically, at time step *t*, the model combines these two sources of information inside the GRU cell as part of the same hidden-state transition. This design should not be interpreted as online hypergraph updating. The hypergraph incidence matrix is constructed offline from the training-window data and is then reused without modification during the subsequent forecasting phase. In this way, offline structure identification is separated from recurrent inference, which helps maintain structural stability, computational efficiency, and chronological consistency in forecasting.

**Figure 3:**
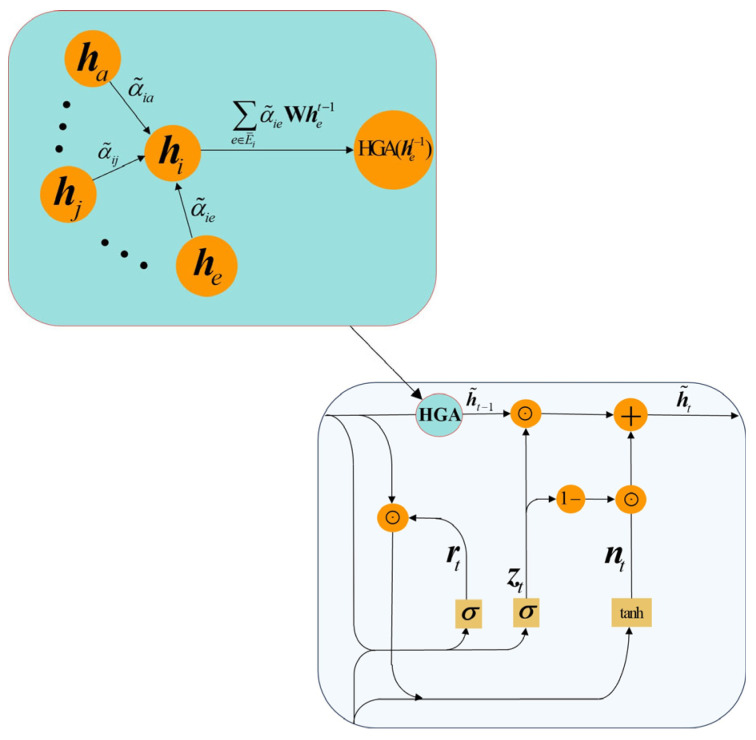
The RST-HGA-GRU structure.

The RST-HGA-GRU contains a hypergraph attention gate that enables in-cell coordination of spatial and temporal information during hidden-state updating. Figure 3 illustrates the internal structure of the RST-HGA-GRU. Firstly, the RST-HGA-GRU accepts the current stock price feature (***x****_t_*) and the hidden feature of the previous moment (***h****_t_*_−1_), and inputs them into Equations (11) and (12) to compute the corresponding reset gate feature and update gate feature, respectively; similarly, the computed reset gate feature, update gate feature, and ***x****_t_* and ***h****_t_*_−1_ are inputted together into Equation (13) to compute the candidate hidden feature (***n****_t_*) of the current unit. Secondly, the spatial incidence matrix (***H***) of the stock hypergraph constructed in Section 3.3 as well as the hidden feature (***h****_t_*_−1_) of the previous moment are inputted into Equations (8) and (9) to compute the attentional weight between the stock and the corresponding hyperedge and normalized using the Softmax function. Next, the computed attentional weight coefficients (${\tilde{\alpha }}_{ie}$) and the hyperedge features of the previous moment ($h_{e}^{t-1}$) are inputted into Equation (10) to compute the historical hidden features (${\tilde{h}}_{i}^{t-1}$) of stock *i* incorporating the spatiotemporal features, reformulating this process as Equation (15). Finally, the historical hidden features (${\tilde{h}}_{i}^{t-1}$) incorporating the spatiotemporal features along with the current stock price features (***x****_t_*) are inputted into Equation (14) to compute the hidden features (${\tilde{h}}_{t}$) of the current cell, reformulating this process as Equation (16). In a sequential loop, the other units of the RST-HGA-GRU network are executed with the same computational process for that unit, and the hidden features of the last unit of the RST-HGA-GRU network are inputted into the fully connected layer to obtain the final stock price prediction.

(15) $${\tilde{h}}_{i}^{t-1}=\mathrm{HGA}(h_{e}^{t-1})=\sum_{e\in {\bar{E}}_{i}}{\tilde{\alpha }}_{ie}Wh_{e}^{t-1}$$

(16) $${\tilde{h}}_{t}=\left(1-z_{t}\right)⊙n_{t}+z_{t}⊙{\tilde{h}}_{i}^{t-1}$$

#### 3.4.4. Recurrent Integration of Hypergraph Relational Aggregation

In the proposed RST-HGA-GRU, hypergraph relational aggregation is incorporated into the recurrent updating process. The hidden state from the previous time step is used not only to preserve temporal memory but also to support higher order relational aggregation over the stock hypergraph. The resulting relation-aware representation then enters the gate-controlled hidden state update of the current step. Through this design, temporal dynamics and cross-stock dependence are coupled within the same recurrent transition, which provides a compact way to model spatiotemporal interaction in stock forecasting.

This distinction is important from a modeling perspective. In many tandem architectures, temporal and spatial dependencies are processed in separate stages. A temporal module first extracts sequential features, and a graph or hypergraph module then refines these features, or vice versa. Although such designs can improve representation quality, the interaction between temporal memory and relational information remains partially decoupled. In contrast, the proposed RST-HGA-GRU performs spatiotemporal coupling inside a single recurrent unit. The previous hidden state is used not only for temporal recursion but also for hypergraph-based relational aggregation, and the resulting spatially informed representation is injected into the gate-controlled update of the current hidden state. Therefore, the recurrent evolution is directly modulated by higher-order cross-stock dependence rather than by a post hoc spatial feature transformation.

A second difference lies in the role of the hypergraph itself. In many existing stock forecasting studies, the relation structure is either predefined from prior knowledge or constructed from pairwise similarity only. In this study, the hypergraph is derived from heterogeneous financial data through tensor decomposition, similarity estimation, and sparsification. As a result, the hypergraph serves as a structured relational prior that reflects both pairwise affinity and higher-order group interactions among stocks. This design enables the recurrent unit to operate on a richer stock dependence structure than ordinary pairwise graph models.

A third clarification concerns the operational scope of the proposed fusion mechanism. The model does not update the hypergraph online when new observations arrive. Instead, the hypergraph is constructed offline from the available training window and then reused during forecasting. Accordingly, the proposed framework should be understood as performing in-cell spatiotemporal fusion during recurrent inference, rather than dynamic graph reconstruction.

Taken together, the methodological novelty of RST-HGA-GRU lies not in introducing a new standalone hypergraph learning theory, but in designing a recurrent forecasting architecture where higher-order relational aggregation is integrated into the state transition itself. This structural difference distinguishes the proposed model from conventional sequential hypergraph plus recurrent forecasting pipelines.

#### 3.4.5. Effect of In-Cell Hypergraph Aggregation on Recurrent Updating

The recurrent update in the proposed RST-HGA-GRU is governed by Equations (15) and (16). Equation (15) generates the fused historical hidden representation ${\tilde{h}}_{i}^{\;t-1}$ through hypergraph attention aggregation over the stock hypergraph, while Equation (16) incorporates this relation-aware representation into the gate-controlled update of the current hidden state. Under this formulation, the previous hidden representation affects the current recurrent update through two coupled pathways. One pathway follows the standard GRU mechanism through the update gate $z_{t}$ and the candidate hidden state $n_{t}$, thereby preserving temporal memory and sequential dependence. The other pathway aggregates the previous hyperedge hidden features induced by the previous node hidden states and yields the relation-aware representation ${\tilde{h}}_{i}^{\;t-1}$ through hypergraph attention aggregation, so that higher-order stock dependence enters the recurrent state transition directly.

This design enriches the role of relational information in recurrent forecasting. In many sequential spatiotemporal architectures, spatial relation modeling is implemented as a separate transformation before or after temporal modeling, and the temporal module then evolves mainly according to its own hidden-state recursion. In the present framework, however, the fused representation ${\tilde{h}}_{i}^{\;t-1}$ participates directly in Equation (16). As a result, the current hidden state is influenced not only by the temporal trajectory of an individual stock but also by the shared structural context encoded by the hypergraph. This tighter coupling is particularly relevant to stock forecasting, where price movements are often shaped by common sectoral, style-related, and market-wide dependence.

The recurrent placement of hypergraph aggregation is also relevant to multi-step forecasting. Because the hidden state is propagated through time, the relation-aware information introduced through ${\tilde{h}}_{i}^{\;t-1}$ can continue to affect subsequent updates along the recurrent chain. In this sense, higher-order stock dependence is not treated as a one-time auxiliary correction, but becomes part of the evolving hidden representation. Moreover, since $z_{t}\in {\left(0,1\right)}^{d}$, Equation (16) shows that ${\tilde{h}}_{t}$ is an element-wise convex combination of $n_{t}$ and ${\tilde{h}}_{i}^{\;t-1}$. Therefore, the model adaptively determines, for each hidden dimension, how much newly generated temporal information should be incorporated and how much structurally aggregated historical information should be retained.

The above mechanism also admits a basic boundedness property. Since the candidate hidden state $n_{t}$ is generated through a tanh activation, each element of $n_{t}$ lies in [−1,1]. Therefore,

(17) $$\|{\tilde{h}}_{t}{\|}_{\infty }\leq \|(1-z_{t})⊙n_{t}{\|}_{\infty }+\|z_{t}⊙{\tilde{h}}_{i}^{\;t-1}{\|}_{\infty }\leq 1+\|{\tilde{h}}_{i}^{\;t-1}{\|}_{\infty }$$

In addition, because the attention coefficients in Equation (15) are SoftMax-normalized, the aggregation stage forms a weighted combination of hyperedge features. Under bounded inputs and finite model parameters, ${\tilde{h}}_{i}^{\;t-1}$ remains bounded on bounded domains. Hence, if the initial hidden state is bounded, the hidden-state sequence remains bounded for all time steps. This indicates that embedding hypergraph aggregation into the recurrent transition preserves the basic bounded behavior expected from gated recurrent models.

A further implication is local continuity and perturbation control. Consider two previous hidden states that generate corresponding quantities $\left(z_{t},n_{t},{\tilde{h}}_{i}^{\;t-1},{\tilde{h}}_{t}\right)$ and $\left({z^{\prime }}_{t},{n^{\prime }}_{t},{{\tilde{h}}_{i}^{\;t-1}}^{\prime },{{\tilde{h}}_{t}}^{\prime }\right)$ under the same input. From Equation (16), their difference can be written as

(18) $${\tilde{h}}_{t}-{\tilde{h}}_{t^{\prime }}=(z_{t^{\prime }}-z_{t})⊙n_{t}+(1-z_{t^{\prime }})⊙(n_{t}-n_{t^{\prime }})+(z_{t}-z_{t^{\prime }})⊙{\tilde{h}}_{i}^{\;t-1}+z_{t^{\prime }}⊙({\tilde{h}}_{i}^{\;t-1}-{\tilde{h}}_{i}^{\;t-1\prime })$$

Under a fixed input $x_{t}$ and fixed hypergraph structure **H**, the quantities $z_{t}$, $n_{t}$, and ${\tilde{h}}_{i}^{\;t-1}$ can all be regarded as functions of the previous node hidden state $h_{t-1}$ on bounded domains. Since affine mappings, Softmax normalization, sigmoid activation, tanh activation, and element-wise multiplication are continuous on bounded domains, there exists a constant C>0 such that

(19) $$\|{\tilde{h}}_{t}-{\tilde{h}}_{t^{\prime }}{\|}_{2}\leq C\|h_{t-1}-h_{t-1^{\prime }}{\|}_{2}$$

Thus, the one-step hidden-state mapping is locally Lipschitz continuous with respect to the previous hidden state on bounded domains. Although this result does not imply global contraction or asymptotic stability under arbitrary parameter settings, it shows that small perturbations in the previous hidden state lead to controlled changes in the updated state. Therefore, the proposed recurrent unit remains well defined and locally well behaved while incorporating higher-order relational aggregation into hidden-state evolution.

#### 3.4.6. Loss Function

The model is trained by minimizing the loss in Equation (20), which consists of a prediction error term and an *L*_2_ regularization term. The first term measures the discrepancy between predicted and actual values, whereas the second term penalizes excessive parameter magnitude to reduce overfitting. The hyperparameter λ controls the trade-off between data fitting and regularization.

(20) $$loss={\left\|X-\hat{X}\right\|}^{2}+\lambda L_{reg}$$

#### 3.4.7. Pseudocode for the RST-HGA-GRU Model

The training process of the RST-HGA-GRU model is summarized in Algorithm 1. This model integrates the HGA module into the GRU to jointly capture dynamic spatial relationships and long-term temporal dependencies in stock data. The HGA module models high-order, evolving interactions among stocks via hypergraph-based attention, while the GRU learns sequential patterns from historical price data. At each time step, the model computes the GRU’s reset gate, update gate, and candidate hidden state. Concurrently, attention weights between stocks and their hypergraph neighbors are calculated to aggregate spatial information. The spatial context is then fused with the temporal candidate state to produce the updated hidden state. A fully connected layer generates predictions, and model parameters are optimized through backpropagation based on the defined loss. By combining temporal modeling with high-order spatial reasoning, the RST-HGA-GRU model enhances spatiotemporal representation learning and improves forecasting accuracy in complex financial markets.
**Algorithm 1** The framework of RST-HGA-GRU**Input: *X***: historical stock price data            *epoch*: maximum number of model training epochs            *iter*: maximum number of time steps per epoch             ***H***: spatial hypergraph (batched)            ***h****_t_*_−1_: previous hidden state **Output:** trained model parameters 1: function TRAIN_RST_HGA_GRU_MODEL (***X***, *epoch*, *iter*, ***H***, ***h****_t_*_−1_) 2:    Initialize model parameters 3:    **for** *i* = 1 to *epoch* **do** 4:        **for** *j* = 1 to *iter* **do** 5:            Calculate reset gate (***r****_t_*), update gate (***z****_t_*) and candidate hidden state (***n****_t_*) based on Equations (11)–(13). 6:            Compute and normalize attention weights $\left({\tilde{\alpha }}_{ie}\right)$ between stocks and their neighbors based on Equations (8) and (9). 7:            Calculate the fused hidden representation $({\tilde{h}}_{i}^{t-1}$$)\;\mathrm{using}\;\mathrm{the}\;\mathrm{previous}\;\mathrm{hyperedge}\;\mathrm{feature}\;(h_{e}^{t-1}$$)\;\mathrm{and}\;\mathrm{spatial}\;\mathrm{attention}\;({\tilde{\alpha }}_{ie}$) via Equation (15). 8:            Update hidden state $({\tilde{h}}_{t}$$)\;\mathrm{using}\;\mathrm{candidate}\;\mathrm{hidden}\;\mathrm{state}\;(n_{t})\;\mathrm{and}\;\mathrm{fused}\;\mathrm{hidden}\;\mathrm{state}\;({\tilde{h}}_{i}^{t-1}$) via Equation (16). 9:            Compute prediction using a fully connected layer. 10:          Calculate loss based on Equation (20). 11:          Update model parameters using backpropagation. 12:        **end for** 13:    **end for** 14:    **return** trained model parameters 15: **end** function

## 4. Experiments

### 4.1. Evaluation Metrics

To evaluate predictive performance, six metrics are used: Root Mean Square Error (*RMSE*), Mean Absolute Error (*MAE*), Mean Absolute Percentage Error (*MAPE*), a normalized accuracy score (*Acc*), the coefficient of determination (*R*^2^), and the explained variance score (*var*). Among them, *Acc* is derived from the relative deviation between predicted and actual values, and higher values indicate better predictive consistency. *RMSE*, *MAE*, and *MAPE* quantify prediction errors, where lower values indicate better performance. In contrast, *R*^2^ and *var* measure the extent to which the predicted values explain the actual observations, with higher values indicating stronger explanatory power. The specific definitions of these metrics are provided below.

(1) Root Mean Square Error (*RMSE*)

(21) $$RMSE=\sqrt{\frac{1}{MN}\overset{M}{\underset{j=1}{\sum }}\overset{N}{\underset{i=1}{\sum }}{\left(y_{i}^{j}-{\hat{y}}_{i}^{j}\right)}^{2}}$$

(2) Mean Absolute Error (*MAE*)

(22) $$MAE=\frac{1}{MN}\overset{M}{\underset{j=1}{\sum }}\overset{N}{\underset{i=1}{\sum }}|y_{i}^{j}-{\hat{y}}_{i}^{j}|$$

(3) Mean Absolute Percentage Error (*MAPE*)

(23) $$MAPE=\frac{1}{MN}\overset{M}{\underset{j=1}{\sum }}\overset{N}{\underset{i=1}{\sum }}\left|\frac{y_{i}^{j}-{\hat{y}}_{i}^{j}}{y_{i}^{j}}\right|$$

(4) Accuracy (*Acc*)

(24) $$Acc=1-\frac{\|Y-\hat{Y}{\|}_{F}}{\|Y{\|}_{F}}$$

(5) Coefficient of Determination (*R*^2^)

(25) $$R^{2}=1-\frac{\sum_{j=1}^{M}\sum_{i=1}^{N}{\left(y_{i}^{j}-{\hat{y}}_{i}^{j}\right)}^{2}}{\sum_{j=1}^{M}\sum_{i=1}^{N}{\left(y_{i}^{j}-\bar{Y}\right)}^{2}}$$

(6) Explained Variance Score (*var*)

(26) $$var=1-\frac{Var\{Y-\hat{Y}\}}{Var\{Y\}}$$

Let $y_{i}^{j}$ and ${\hat{y}}_{i}^{j}$ represent the real and predicted values of stock *i* at prediction interval *j*, respectively. Let *M* and *N* denote the number of stocks and time steps, respectively. Y and $\hat{Y}$ denote the sets of actual and predicted values, and $\bar{Y}$ represents the mean of Y. *RMSE*, *MAE* and *MAPE* are used to quantify prediction error, where smaller values indicate better accuracy. In contrast, *R*^2^, *Acc*, and *var* measure how well the predicted values explain the actual data, with higher values reflecting stronger predictive performance.

### 4.2. Benchmark Models

To evaluate the effectiveness of the proposed RST-HGA-GRU model, its predictive performance is compared with the following benchmark models:

(1) ARIMA model [2]: The Autoregressive Integrated Moving Average (ARIMA) model is a classical statistical forecasting method that models temporal dependence through autoregressive and moving-average terms. It is used here as a conventional univariate time-series baseline for short-horizon stock forecasting.
(2) RFR model [22]: The Random Forest Regression (RFR) model is an ensemble learning method that builds multiple decorrelated decision trees and aggregates their outputs for regression. It is included as a representative machine-learning baseline with nonlinear fitting ability.
(3) SVR model [23]: Support Vector Regression (SVR) extends the support vector machine framework to continuous prediction tasks by learning an optimal regression hyperplane in a transformed feature space. It is used as a representative kernel-based regression baseline.
(4) LSTM model [28]: The Long Short-Term Memory (LSTM) network employs gated memory units to regulate information flow and capture long-range temporal dependence. It is included as a standard deep-learning baseline for sequential forecasting.
(5) GRU model [31]: The Gated Recurrent Unit (GRU) is a simplified gated recurrent architecture that uses update and reset gates to control hidden-state evolution. Owing to its compact structure and competitive forecasting ability, it is used as the basic recurrent baseline in this study.
(6) ST-GCN-GRU model [9]: This model is a tandem spatiotemporal baseline in which graph convolution is first applied for spatial feature aggregation and the resulting representations are then passed to a GRU for temporal modeling. Its design follows the common sequential spatial-then-temporal paradigm adopted in prior spatiotemporal stock forecasting studies.
(7) ST-GAT-GRU model [10]: This model is also a tandem spatiotemporal baseline, but replaces graph convolution with graph attention so that neighboring stock information can be aggregated with adaptive importance weights before GRU-based temporal modeling. It is included to represent the sequential attention-based graph forecasting paradigm reported in related studies.
(8) ST-HGA-GRU model: The Spatiotemporal Hypergraph Attention GRU (ST-HGA-GRU) model is a tandem spatiotemporal baseline in which hypergraph attention is used for spatial aggregation and the resulting spatial representations are then fed into a GRU for temporal modeling. This sequential spatial-then-temporal design is close in spirit to prior hypergraph-based stock forecasting architectures such as DHSTN [11], while being implemented here as a comparative baseline under the unified experimental setting of this study.
(9) RST-GCN-GRU model: This model is an internal variant of the proposed framework, obtained by replacing the hypergraph attention module in RST-HGA-GRU with graph convolution. It is introduced to examine whether the recurrent fusion framework itself remains effective when higher-order hypergraph aggregation is replaced by pairwise graph convolution.
(10) RST-GAT-GRU model: This model is another internal variant of the proposed framework, obtained by replacing the hypergraph attention module in RST-HGA-GRU with graph attention. It is used to compare the proposed hypergraph-based recurrent fusion design with a graph-attention-based counterpart under the same recurrent integration mechanism.

### 4.3. Hyperparameter Settings

The hyperparameters of the proposed RST-HGA-GRU are summarized in Table 2, including those associated with the HGA module, the GRU module, and the general training process. For the HGA module, the main hyperparameters include the number of input channels (in_channels), hidden feature dimension (hid_dim), number of output channels (out_channels), dropout rate (drop_rate), negative slope of the Leaky ReLU activation (atten_neg_slope), and the number of attention heads (num_heads). For the GRU module, the hyperparameters include the input feature size (input_size), hidden state size (hidden_size), output size (out_size), and the number of GRU layers (gru_layers). In addition, the training-related hyperparameters include the optimizer, number of training epochs (epoch), input sequence length (input_len), prediction horizon (pre_len), batch size (batch_size), learning rate, and weight decay. The final selected hyperparameter values are reported in the last column of Table 2.

In this study, the hyperparameters of RST-HGA-GRU were determined by a validation-based empirical tuning procedure. Specifically, candidate values were specified for each hyperparameter, and different combinations were tested under the same experimental protocol. The validation loss was then used as the model selection criterion, and the hyperparameter combination achieving the best validation performance was chosen as the final setting. This strategy allows the model configuration to be adjusted according to empirical performance while maintaining a transparent and reproducible selection process. It should be noted that, due to architectural constraints, the numbers of input channels (in_channels) and output channels (out_channels) in the HGA module must be consistent with the hidden state size (hidden_size) in the GRU module. In addition, the dataset was divided into training and validation subsets with a ratio of 8:2 during hyperparameter selection. To ensure fair comparison, all benchmark models were evaluated under the same rolling-window sample generation scheme, direct multi-step forecasting setting, and chronological training-validation split. Model-specific hyperparameters were selected separately for each benchmark according to validation performance under the same experimental protocol.

For horizon-specific experiments, pre_len was set to the target horizon (1, 2, 3, 4, 5, 6). Table 2 reports the base configuration, while the remaining hyperparameters were kept unchanged across horizons unless otherwise stated. To ensure a fair comparison, all models were trained and evaluated under the same experimental protocol, including the chronological data split, rolling-window sample generation scheme, input sequence length, forecasting horizon, and model selection criterion. Multi-step forecasting was implemented in the direct setting for all methods, so that each model predicted the target horizon in a single forward pass rather than by recursive iteration. Model-specific hyperparameters were selected separately for each baseline according to validation performance under the same experimental protocol. At the same time, all baselines were tuned and selected on the same validation period, using the same data split and forecasting targets, so that performance differences cannot be attributed to unequal information access or inconsistent evaluation settings. For graph-based and hypergraph-based baselines, the corresponding spatial structures were constructed from the training window only and then reused in the associated validation phase. For models without spatial modules, the same node-wise temporal inputs and the same forecasting targets were used. In this way, the comparison isolates the effect of model architecture as much as possible while keeping the sample generation and evaluation pipeline consistent across methods. In addition, the same maximum training epoch budget and the same early-stopping strategy were used across the deep learning models.

To further strengthen the competitiveness of the tandem spatiotemporal baselines, an additional hyperparameter optimization procedure was conducted for ST-GCN-GRU, ST-GAT-GRU, and ST-HGA-GRU. Specifically, Optuna was used to search the main architecture-related and optimization-related hyperparameters under the same chronological split, validation period, epoch budget, and early stopping rule as those adopted for the proposed framework. The corresponding search spaces are summarized in Table 3. This additional tuning step was introduced to reduce the possibility that the weak performance of the tandem baselines was caused by limited hyperparameter exploration rather than by model formulation.

### 4.4. Results and Analysis

#### 4.4.1. Comparison with Benchmark Models

Under the unified forward-looking protocol described in Section 4.3, Table 4 reports the benchmark results across multiple forecasting horizons and evaluation metrics. The results show that the proposed RST-HGA-GRU consistently outperforms the conventional statistical, machine learning, and tandem spatiotemporal baselines, while also achieving the best overall performance within the recurrent-fusion model family.

Table 4 presents the prediction performance of the ten benchmark models and the proposed RST-HGA-GRU model for 1-day, 2-day, and 3-day forecasts, evaluated across multiple metrics. The results demonstrate that the RST-HGA-GRU model consistently outperforms all benchmark models in predicting stock closing prices across the 1-day, 2-day, and 3-day forecast horizons reported in Table 4. This superior performance across multiple evaluation metrics and time intervals underscores the robustness and accuracy of the proposed model. As shown in Table 4, neural network-based models exhibit stronger capabilities in capturing temporal dependencies compared to traditional machine learning models. Although the LSTM model slightly outperforms the GRU model due to its more complex gating mechanism, the GRU offers advantages in terms of fewer parameters and faster training, making it more efficient for deep learning applications. Given these advantages, the GRU is widely adopted in both existing hybrid models (ST-GCN-GRU, ST-GAT-GRU, ST-HGA-GRU) and the proposed embedded recurrent spatiotemporal fusion models (RST-GCN-GRU, RST-GAT-GRU, and RST-HGA-GRU), enhancing their ability to model temporal features effectively.

An additional observation from Table 4 is that the tandem spatiotemporal baselines, including ST-GCN-GRU, ST-GAT-GRU, and ST-HGA-GRU, perform substantially worse than both the temporal-only baselines and the proposed RST-type models under the same chronological split, rolling-window sample generation process, direct multi-step forecasting setting, and validation-based model selection protocol. A plausible explanation is that the tandem architectures perform spatial transformation and temporal updating in separate stages, which may weaken the preservation of raw temporal dynamics when the stock relation structure is noisy, dense, or only partially aligned with short-horizon price evolution. By contrast, the proposed RST-type models inject relational aggregation directly into the recurrent update, allowing temporal memory and cross-stock dependence to interact within the same state transition.

Furthermore, when comparing embedded spatiotemporal fusion models, the RST-GAT-GRU model, using GAT for spatial feature extraction, achieves a 9.14% lower *RMSE* on average than the RST-GCN-GRU model, indicating that the GAT’s attention-based differential aggregation of spatial correlations is more effective than the uniform aggregation of GCN. Notably, the RST-HGA-GRU model, which leverages a hypergraph attention mechanism to extract higher-order spatial correlations, achieves a further 10.21% reduction in *RMSE* compared to RST-GAT-GRU. This highlights the superiority of hypergraph structures in capturing complex, high-order relationships among stocks over conventional pairwise-based graph methods.

Overall, the embedded recurrent spatiotemporal fusion models (RST-GCN-GRU, RST-GAT-GRU, and RST-HGA-GRU) show more competitive overall performance than the temporal-only models across the reported settings, demonstrating the value of incorporating relational information into recurrent forecasting.

#### 4.4.2. Additional Validation of Tandem Spatiotemporal Baselines

To further examine whether the weak performance of the tandem spatiotemporal baselines is sensitive to random initialization, we conducted repeated-run validation under three different random seeds after fixing the Optuna-selected hyperparameters. In this analysis, ST-GCN-GRU, ST-GAT-GRU, and ST-HGA-GRU were retrained under the same data split, training budget, and evaluation protocol, and the mean and standard deviation of the main metrics were recorded. The results are reported in Table 5.

Table 5 shows that the relative ranking of the tandem spatiotemporal baselines remains stable across random seeds and forecasting horizons. ST-GAT-GRU consistently achieves the best average performance among the three tandem baselines, whereas ST-HGA-GRU remains the weakest model in nearly all reported metrics. Although some variation across seeds can be observed, especially for ST-GAT-GRU at longer horizons, the overall ordering is preserved throughout the 1- to 6-day forecasting range. These findings indicate that the weak performance of the tandem baselines cannot be attributed solely to an unfavorable single random seed. In particular, ST-HGA-GRU exhibits very small standard deviations while still producing the worst average results, which suggests that its inferior performance is stable rather than accidental. Together with the persistently negative *R*^2^ values and weak explained-variance scores, this result further supports the view that, under the present forecasting setting, sequential spatial-then-temporal coupling is less effective than tighter recurrent fusion.

#### 4.4.3. Multi-Step Forecast Analysis

**Figure 4:**
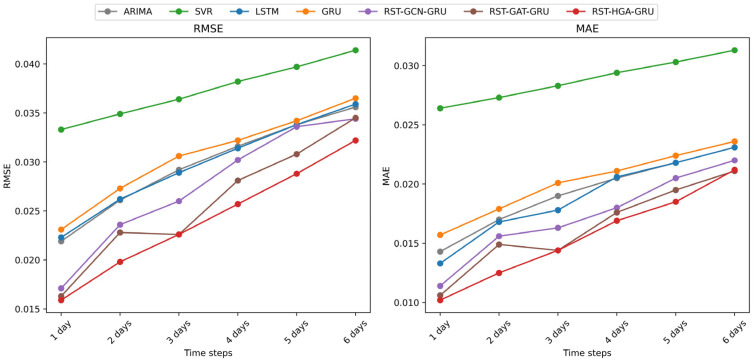
Trend of *RMSE* and *MAE* for multi-step prediction of models.

**Figure 5:**
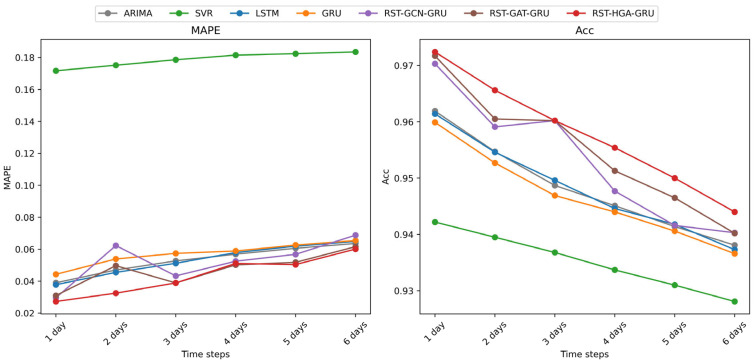
Trend of *MAPE* and *Acc* for multi-step prediction of models.

**Figure 6:**
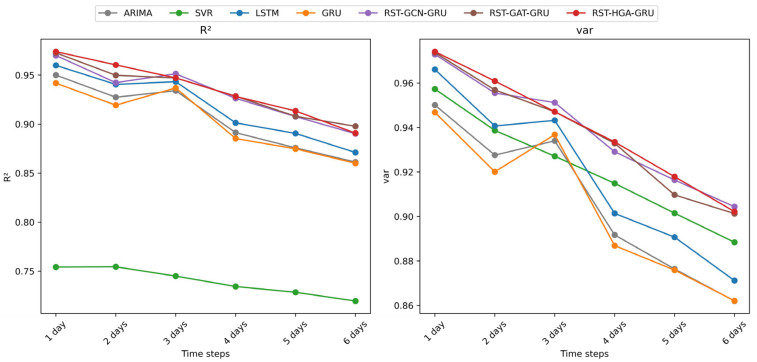
Trend of *R*^2^ and *var* for multi-step prediction of each model.

To further evaluate the performance of different models in multi-step forecasting tasks, we analyzed the trends of six metrics, *RMSE*, *MAE*, *MAPE*, *Acc*, *R*^2^, and *var*, under different forecasting horizons ranging from 1 to 6 days, as illustrated in Figure 4, Figure 5 and Figure 6. For readability, Figure 4, Figure 5 and Figure 6 show representative temporal baselines together with the three RST variants, while the complete numerical comparisons for all models are reported in Table 4.

As shown in Figure 4, both *RMSE* and *MAE* exhibit a consistent upward trend as the prediction step increases, which reflects the increasing difficulty of long-horizon prediction. Among the representative models shown in Figure 4, Figure 5 and Figure 6, SVR performs the worst across most forecasting horizons. In contrast, deep learning-based models such as LSTM and GRU provide more competitive results due to their capability to capture temporal patterns. Nevertheless, these models still fall short when compared to the graph-enhanced models. Specifically, the RST-GCN-GRU, RST-GAT-GRU, and RST-HGA-GRU models, which integrate graph and hypergraph spatial dependencies into temporal learning, demonstrate clear advantages. Among them, the RST-HGA-GRU consistently achieves the lowest *RMSE* and *MAE* across all time steps, indicating superior prediction precision and robustness, especially at longer horizons within the tested range.

Figure 5 presents the variations in *MAPE* and *Acc* over the same forecast horizons. A similar trend is observed, where SVR again yields the poorest performance, with relatively high *MAPE* and declining accuracy as the number of prediction steps increases. On the other hand, all deep learning models outperform SVR, with RST-HGA-GRU maintaining the lowest *MAPE* values and the highest prediction accuracy throughout. This result highlights the model’s ability to effectively reduce relative prediction error while retaining high predictive consistency under the normalized accuracy measure, even as forecasting complexity grows. The performance gap becomes more prominent as the prediction horizon increases, showcasing the advantages of incorporating higher-order topological relationships via the hypergraph mechanism.

In Figure 6, *R*^2^ and *var* metrics provide further insights into model interpretability and prediction variance retention. SVR again underperforms in terms of *R*^2^, showing limited capability in explaining the variation in target variables. Deep learning-based models exhibit better fit, while the graph-augmented models—particularly RST-HGA-GRU—achieve consistently higher *R*^2^ values, exceeding 0.95 at short horizons and remaining superior even with longer forecasts. Meanwhile, the *var* of most models tends to decrease with increasing time steps, reflecting a typical over-smoothing phenomenon in long-term prediction. However, RST-HGA-GRU exhibits a slower rate of variance decay, thereby better preserving the volatility characteristics of the original financial time series.

Overall, the RST-HGA-GRU model demonstrates superior performance across all evaluation metrics in multi-step forecasting scenarios. Its ability to maintain low prediction error, high accuracy, strong explanatory power, and variance retention suggests that the integration of hypergraph structures and attention mechanisms is highly effective in capturing complex spatiotemporal dependencies in financial data. These findings reinforce the model’s practical value in multi-step financial forecasting, particularly when predictive accuracy and stability over longer horizons within the tested range are important.

#### 4.4.4. Sensitivity Analysis of Hypergraph Construction Parameters

Since the hypergraph construction in Section 3.3 depends on both the Top-K sparsification parameter and the Tucker ranks selected by PCA, we further evaluate the robustness of the proposed framework with respect to these two design choices.

Specifically, two sensitivity analyses are conducted. The first investigates the effect of the Top-K parameter K, which determines how many of the most similar stocks are used to form each hyperedge. The second examines the effect of the PCA threshold used to determine the Tucker ranks (*R_t_*, *R_n_*, *R_f_*) along the temporal, stock, and feature modes. Here, the threshold denotes the cumulative explained variance retained in PCA and therefore directly controls the compactness of the Tucker representation.

(1) Sensitivity to the Top-K parameter

**Figure 7:**
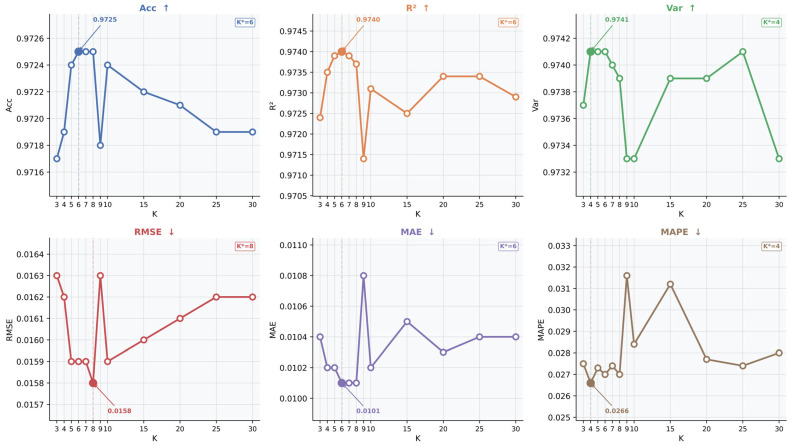
Sensitivity of forecasting performance to the Top-K parameter in hypergraph construction.

To evaluate the influence of local neighborhood size in hypergraph construction, *K* is varied from 3 to 30 while keeping the other settings unchanged, and the corresponding results are shown in Figure 7. Overall, the proposed model exhibits relatively stable performance over a moderate range of *K*, which indicates that the hypergraph construction is not overly sensitive to a narrowly tuned neighborhood size. In particular, the model achieves consistently strong performance when *K* lies approximately between 4 and 8, where *Acc*, *R*^2^, and *var* remain high and *RMSE* and *MAE* remain low.

(2) Sensitivity to the PCA threshold for Tucker rank selection

**Figure 8:**
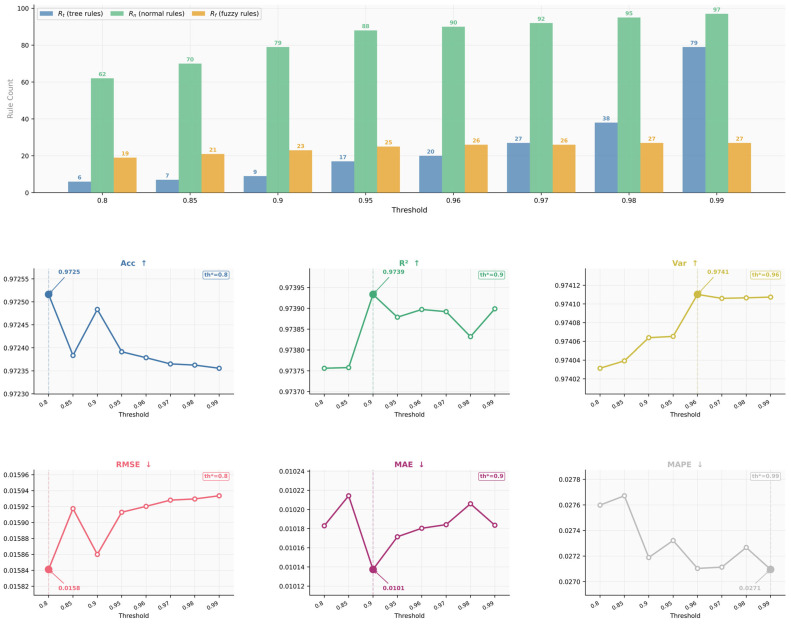
Sensitivity of forecasting performance and Tucker ranks to the PCA threshold for Tucker rank selection.

We next analyze the effect of the PCA threshold used to determine the Tucker ranks. The threshold is varied from 0.80 to 0.99, with *K* fixed at 5. The resulting Tucker ranks and forecasting performance are shown in Figure 8. As the threshold increases, more variance is retained in each tensor mode, and the selected Tucker ranks grow accordingly. Specifically, (*R_t_*, *R_n_*, *R_f_*) increase from (6, 62, 19) at a threshold of 0.80 to (79, 97, 27) at a threshold of 0.99. This confirms that the threshold directly controls the complexity of the latent representation used for hypergraph construction.

From the performance curves, the proposed model remains overall stable across the tested thresholds, indicating that the tensor decomposition-based hypergraph construction is not highly sensitive to a specific PCA threshold. Some lower thresholds achieve slightly better values on individual metrics. For example, threshold (0.80) gives the highest *Acc* and the lowest *RMSE*, while threshold (0.90) yields the best *R*^2^ and *MAE*. However, these advantages are marginal, and the overall performance differences among thresholds from 0.90 to 0.99 remain small.

In this study, threshold (0.95) is adopted as the default setting for Tucker rank selection for three reasons. First, retaining 95% explained variance is a widely used and relatively conservative PCA criterion, which helps preserve the dominant latent structure of the original tensor while avoiding overly aggressive compression. Second, threshold (0.95) maintains stable forecasting performance across all metrics, indicating that it does not lead to a substantial loss of predictive information. Third, compared with higher thresholds such as 0.96, 0.97, 0.98, and 0.99, threshold (0.95) avoids further growth of the Tucker ranks, especially along the temporal and stock modes, without sacrificing meaningful predictive accuracy. Under this setting, the selected Tucker ranks are (17, 88, 25), which provide a balanced compromise between information retention, representation compactness, and predictive stability.

Overall, the results in Figure 7 and Figure 8 show that the proposed hypergraph construction is reasonably robust to moderate variations in both the Top-K parameter and the PCA threshold used for Tucker rank selection. These findings support the stability of the tensor decomposition-based hypergraph construction strategy adopted in the proposed RST-HGA-GRU framework.

#### 4.4.5. Diebold–Mariano Tests Across Multiple Forecasting Horizons

To further assess whether the predictive gains of the proposed RST-HGA-GRU are statistically meaningful, we extend the Diebold-Mariano (DM) tests from the original one-step setting to exact multi-horizon forecasts from 1 to 6 days ahead. For each horizon h, the test is performed on the exact h-day-ahead prediction errors rather than on the aggregated multi-step output. This design makes the statistical validation directly consistent with the horizon-specific forecasting results reported in Table 4 and in the multi-step analysis.

Two loss functions are considered. The first is the absolute error loss, defined as the absolute difference between the predicted and true stock prices. This loss weights all prediction errors linearly and is relatively robust to occasional large deviations, so it mainly reflects the model’s overall ability to control average prediction error. The second is the squared error loss, defined as the squared difference between the predicted and true values. Since larger deviations receive disproportionately greater penalties under this loss, it is more sensitive to extreme prediction errors and therefore provides a stricter assessment of forecast stability. By examining both losses, the DM analysis can evaluate not only whether the proposed model reduces average error, but also whether it suppresses large forecast deviations more effectively than the benchmark models.

**Figure 9:**
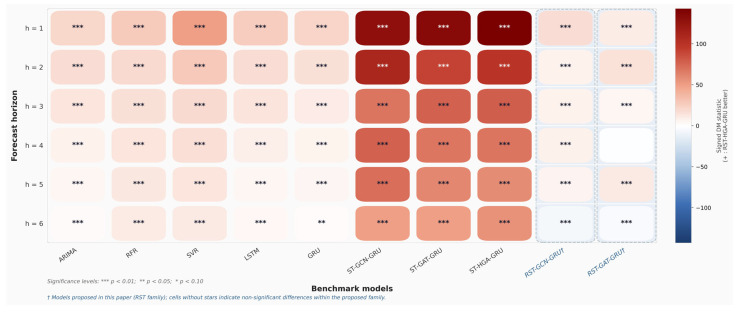
DM test results for exact *h*-day-ahead forecasts under absolute error loss. The heatmap color represents the signed DM statistic, where positive values indicate that RST-HGA-GRU yields lower loss than the corresponding benchmark model. Asterisks denote statistical significance at the 10%, 5%, and 1% levels. The dashed boxes highlight the RST family proposed in this study. Cells without asterisks indicate differences that are not statistically significant at the 10% level.

**Figure 10:**
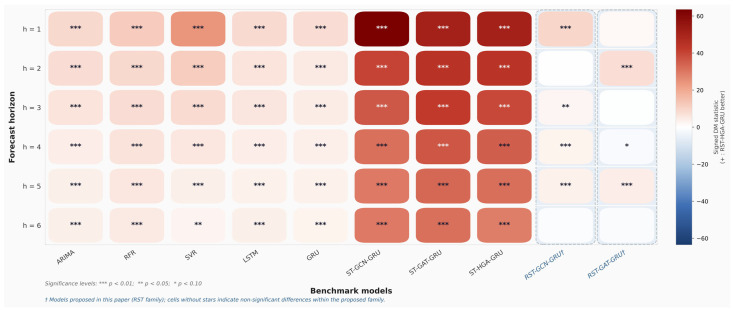
DM test results for exact *h*-day-ahead forecasts under squared error loss. The heatmap color represents the signed DM statistic, where positive values indicate that RST-HGA-GRU yields lower loss than the corresponding benchmark model. Asterisks denote statistical significance at the 10%, 5%, and 1% levels. The dashed boxes highlight the RST family proposed in this study. Cells without asterisks indicate differences that are not statistically significant at the 10% level.

Figure 9 presents the DM test results under the absolute error loss, while Figure 10 reports the corresponding results under the squared error loss. In both figures, the heatmap color represents the signed DM statistic, where a positive value indicates that the proposed RST-HGA-GRU yields lower loss than the corresponding benchmark model. Significance levels are marked by asterisks within each cell. As shown in Figure 9, the proposed RST-HGA-GRU achieves statistically significant improvements over the traditional statistical, machine learning, and tandem spatiotemporal baselines across almost all forecast horizons under absolute error loss. In particular, the advantages over ARIMA, RFR, SVR, LSTM, GRU, ST-GCN-GRU, ST-GAT-GRU, and ST-HGA-GRU are consistently positive and mostly significant at the 1% level. This indicates that the proposed model provides a stable reduction in overall forecast error from short-horizon to longer-horizon prediction. The strongest gains are observed against the tandem spatiotemporal baselines, which is consistent with the performance comparison in Table 4 and further supports the effectiveness of the proposed in-cell spatiotemporal fusion mechanism.

A similar overall pattern is observed in Figure 10 under squared error loss. The proposed model again significantly outperforms the traditional baselines and the tandem spatiotemporal graph baselines over nearly all horizons, indicating that its advantages are not limited to average error reduction but also extend to the suppression of large prediction deviations. This result suggests that the proposed model provides not only more accurate but also more stable forecasts when large errors are penalized more heavily. At the same time, Figure 9 and Figure 10 reveal a more nuanced comparison within the RST family. The performance gap between RST-HGA-GRU and the other two recurrent spatiotemporal fusion variants, namely RST-GCN-GRU and RST-GAT-GRU, is noticeably smaller than the gap against the non-RST baselines. This observation is important because it indicates that a substantial part of the predictive improvement comes from the proposed recurrent spatiotemporal fusion framework itself, that is, from injecting relational aggregation directly into the recurrent state transition rather than using a tandem spatial temporal architecture. Within this unified RST framework, replacing GCN or GAT aggregation with hypergraph attention still yields additional gains, but these gains are more moderate and horizon-dependent.

This distinction is especially clear under squared error loss in Figure 10, where the advantage of RST-HGA-GRU over RST-GAT-GRU becomes weaker at some horizons and is no longer uniformly significant. This suggests that, although hypergraph attention improves overall predictive performance, its incremental benefit over the strongest graph-based RST variant is more evident in general error control than in every large-error case. Such a result is reasonable because the three RST variants share the same in-cell fusion principle and differ mainly in the form of relational aggregation. Therefore, the largest statistical gap is expected between the proposed RST-HGA-GRU and the conventional baselines, whereas the gap within the RST family is naturally smaller.

Overall, the DM evidence from Figure 9 and Figure 10 provides strong statistical support for the proposed method. The results show that the superiority of RST-HGA-GRU is maintained across multiple forecasting horizons and under two complementary loss measures. This strengthens the conclusion that the proposed framework improves both average forecast accuracy and robustness to large prediction errors in stock price forecasting.

#### 4.4.6. Ablation Study

Ablation experiments were conducted to examine the contribution of the main components in the proposed RST-HGA-GRU framework. The model involves two key design aspects. The first is the Tucker-decomposition-based hypergraph construction strategy, which derives a latent relational representation before hypergraph formation. The second is the embedded spatiotemporal fusion mechanism within the recurrent update, where spatial aggregation is incorporated directly into the GRU state transition rather than applied as a separate preprocessing stage. Table 6 reports the results of seven comparative variants together with the full RST-HGA-GRU model across six forecasting horizons. The ST-GCN-GRU, ST-GAT-GRU, and ST-HGA-GRU models rep-resent tandem architectures, whereas RST-GCN-GRU and RST-GAT-GRU retain the recurrent fusion framework but replace hypergraph aggregation with graph convolution or graph attention. In addition, two construction-oriented variants are introduced.

These two variants are used to examine whether the proposed tensor-based hypergraph construction provides advantages over simpler relational structures. (a) RST-HGA-GRU (w/o TD) removes Tucker decomposition and constructs the hypergraph directly from the original stock features using similarity computation and Top-K sparsification. (b) RST-HGA-GRU (Corr-HG) replaces the tensor-based hypergraph with a simpler correlation-based hypergraph constructed from the Pearson similarity matrix of stock returns in the training window, using a correlation threshold of 0.95 and a Top-5 neighborhood rule.

The results in Table 6 reveal a clear performance gap between the tandem baselines and the recurrent-fusion variants. Across all forecasting horizons, ST-GCN-GRU, ST-GAT-GRU, and ST-HGA-GRU perform substantially worse than the corresponding RST-type models, which indicates that applying spatial modeling and temporal updating in separate stages is less effective in the present forecasting task. By contrast, RST-GCN-GRU, RST-GAT-GRU, and RST-HGA-GRU consistently achieve much lower prediction errors and higher explanatory power, supporting the benefit of embedding relational aggregation directly into the recurrent update.

Within the recurrent-fusion family, the proposed RST-HGA-GRU achieves the best or near-best overall performance across the 1- to 6-day forecasting horizons. This result suggests that higher-order hypergraph aggregation remains beneficial after spatial and temporal information are tightly coupled within the recurrent transition. The comparison with RST-HGA-GRU (w/o TD) further shows that removing Tucker decomposition leads to a consistent decline in performance, especially in terms of *RMSE*, *MAPE*, and *R*^2^, which supports the role of latent representation learning in reducing redundancy and noise before hypergraph construction.

The comparison with the correlation-based hypergraph variant provides an additional perspective on the relational construction strategy. Although this simpler structure is more interpretable and remains competitive on several individual metrics, its overall performance is still generally weaker than that of the proposed tensor-based hypergraph. This suggests that the gains of the proposed framework cannot be attributed merely to the use of an arbitrary relational prior. Rather, constructing the hypergraph from Tucker-based latent representations yields a more informative and stable dependence structure than either direct raw-feature similarity or return-correlation-based relations alone.

#### 4.4.7. Simple Gross Back Testing Analysis

To provide a preliminary assessment of practical relevance, a simple gross back testing analysis was conducted based on the one-day-ahead forecasts. On each trading day, stocks were ranked according to their predicted next-day returns implied by the forecasted closing prices, and an equal-weight portfolio was formed by buying the top-10 predicted stocks and holding the position for one trading day. The portfolio was rebalanced daily, and no transaction cost was included in this diagnostic exercise.

**Figure 11:**
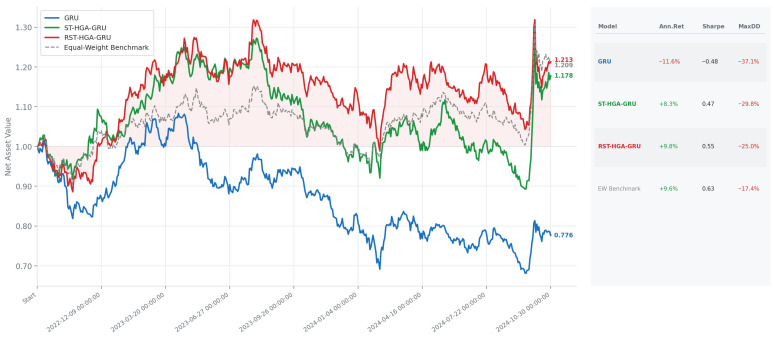
Cumulative gross net asset value curves under the one-day-ahead top-10 long-only strategy.

The cumulative gross net asset value curves are shown in Figure 11. Among the model-based strategies, RST-HGA-GRU achieves the strongest overall performance, with a final net asset value of approximately 1.21, an annualized return of 9.8%, and a Sharpe ratio of 0.55. ST-HGA-GRU remains competitive but yields a lower annualized return of 8.3%, a lower Sharpe ratio of 0.47, and a larger maximum drawdown of 29.8%, while GRU performs substantially worse. This result is broadly consistent with the forecasting comparison, but the performance ordering in back testing does not need to match the error-based results exactly, because point forecasting metrics evaluate price-level approximation, whereas the present back testing exercise depends on cross-sectional stock ranking and the behavior of a specific portfolio rule. In this sense, the gross back testing result reflects not only forecast accuracy, but also how effectively the model transforms predicted prices into useful stock-selection signals.

At the same time, the equal-weight benchmark remains slightly stronger in risk-adjusted terms, with a Sharpe ratio of 0.63 and a smaller maximum drawdown of 17.4%. This is likely related to the simplicity and diversification of the benchmark portfolio, as well as the upward bias of the test-period market environment. Therefore, the result in Figure 11 should be interpreted as a preliminary gross diagnostic rather than evidence of a fully optimized trading strategy. Overall, the back testing evidence suggests that the proposed model contains useful stock-ranking information under a basic one-day-ahead stock-selection setting, even though a simple ranking portfolio does not coincide exactly with the objective measured by conventional forecasting error metrics.

#### 4.4.8. Prediction Results Visualization

**Figure 12:**
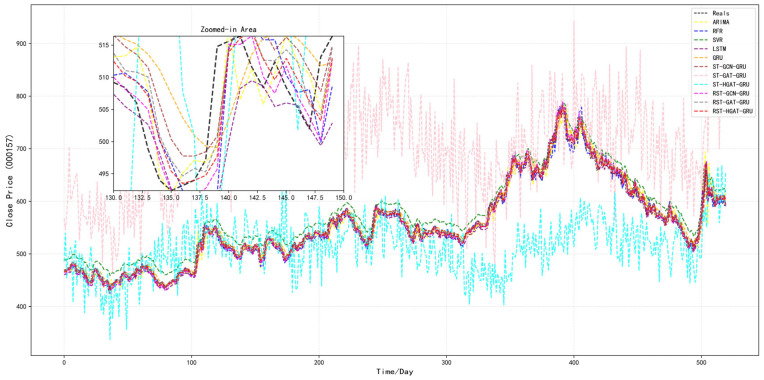
Visualization of prediction results for stock 000157.

To comprehensively evaluate the predictive performance of each model, a stock was randomly selected from the validation set, and a rolling window method was applied to forecast the next day’s closing price. The prediction results are illustrated in Figure 12. Visual inspection reveals that while the traditional statistical model ARIMA captures overall trends, it performs poorly in identifying local extrema. Traditional machine learning models such as RFR and SVR show even greater deviation from actual values, resulting in lower prediction accuracy than ARIMA. Time series-based neural networks, including GRU and LSTM, effectively model temporal dependencies and overall trends but struggle to capture short-term fluctuations and local extrema. This limitation is primarily due to their focus on long-term dependencies, which reduces their sensitivity to abrupt changes.

In contrast, the recurrent-fusion spatiotemporal variants RST-GCN-GRU and RST-GAT-GRU demonstrate improved performance in capturing local extrema. By integrating graph-based spatial aggregation into recurrent updating, these models exploit both spatial and temporal information more effectively than temporal-only baselines. Among the recurrent-fusion spatiotemporal models, the proposed RST-HGA-GRU achieves the best overall tracking performance. This is likely due to lag and insufficient integration in the spatiotemporal fusion process, particularly when spatial features are extracted without recurrent temporal updates, limiting the synergistic effect of spatiotemporal interactions. Meanwhile, the sequential models ST-GCN-GRU, ST-GAT-GRU, and ST-HGA-GRU exhibit the weakest performance. Their tandem fusion structure may impair temporal feature learning by interrupting or distorting temporal dependencies during spatial feature extraction, thereby degrading predictive accuracy.

In summary, the RST-HGA-GRU model outperforms all other models by effectively addressing the limitations of delayed and insufficient fusion in traditional spatiotemporal models. Through its hypergraph attention mechanism and in-cell fusion design, it captures complex, high-order spatiotemporal interactions across multiple dimensions, achieving more accurate one-step prediction tracking and better capture of local fluctuations.

### 4.5. Discussion

The results show that the proposed RST-HGA-GRU provides a consistent advantage for stock price forecasting under the present experimental setting. This advantage comes from two related aspects. First, relational aggregation is embedded directly into the recurrent state transition, so that temporal memory and cross-stock dependence interact within the same hidden-state update rather than being processed in separate stages. Second, within this recurrent fusion framework, hypergraph attention further improves the modeling of higher-order stock dependence. Thus, the gains of RST-HGA-GRU arise from the combination of recurrent spatiotemporal fusion and richer relational representation.

As shown in Table 4, RST-HGA-GRU achieves the best overall performance across the reported metrics and forecast horizons from 1 to 6 days. Although predictive accuracy declines gradually as the horizon increases, which is expected in stock forecasting, the proposed model remains comparatively strong throughout the tested range. Traditional machine learning models such as RFR and SVR are less effective in capturing nonlinear and time-varying dependencies, especially at longer horizons. Temporal deep learning baselines such as LSTM and GRU improve sequential modeling capacity, but they do not explicitly encode cross-stock structural dependence and therefore still provide only a partial description of market dynamics.

The contrast between tandem spatiotemporal baselines and recurrent-fusion models is particularly informative. ST-GCN-GRU, ST-GAT-GRU, and ST-HGA-GRU incorporate spatial information, but spatial transformation and temporal updating are still implemented in separate stages. By comparison, the RST family injects relational aggregation directly into the recurrent update, allowing temporal memory and stock dependence to interact within the same transition step. The large performance gap between the tandem baselines and the RST variants therefore suggests that recurrent spatiotemporal fusion is a major source of improvement. At the same time, the smaller gap within the RST family indicates that the main gain comes first from the recurrent fusion framework itself, while hypergraph attention provides a further but more refined enhancement.

The ablation results in Table 6 support this interpretation. The weaker performance of RST-HGA-GRU (w/o TD) shows that Tucker-decomposition-based latent representation learning improves hypergraph construction beyond direct similarity estimation on the original multi-source features, which can be affected by redundancy, scale heterogeneity, and short-term noise. The comparison with RST-HGA-GRU (Corr-HG) provides an additional perspective. Although the correlation-based hypergraph is simpler and more interpretable, its overall performance remains weaker than that of the full model across most horizons, suggesting that the gains cannot be attributed merely to the use of an arbitrary relational prior.

The sensitivity analyses and multi-horizon DM tests provide further support for the robustness of the framework. The sensitivity results show that the proposed method remains reasonably stable under moderate variations in both the Top-K parameter and the PCA threshold for Tucker rank selection, indicating that the hypergraph construction does not rely on narrowly tuned settings. The DM results further show that the predictive gains of RST-HGA-GRU remain statistically significant across exact 1- to 6-day-ahead forecasts under both absolute error loss and squared error loss. Together, these findings indicate that the proposed framework improves not only average forecast accuracy but also robustness to larger prediction errors.

From a computational perspective, RST-HGA-GRU is moderately more expensive than tandem hypergraph-recurrent architectures because hypergraph-based aggregation is performed inside the recurrent update at each time step. However, the model retains the same asymptotic order as the tandem design and mainly incurs a larger constant factor. In practice, this additional cost primarily affects training, while the hypergraph itself is constructed offline on the training window and then reused during forecasting.

Several limitations should also be noted. Hypergraph construction still depends on feature design, tensor decomposition, PCA-based rank selection, and sparsification choices. Although the sensitivity analyses indicate reasonable robustness within the tested range, the resulting structure is not universally invariant and may vary with the data source, feature definition, or market environment. Scalability may also become a concern as the number of stocks increases, even with sparse implementations. In addition, the current experiments are conducted on a stable subset of CSI 300 constituent stocks over a specific period, so the findings should be interpreted primarily within the current large-cap A-share setting, and broader validation across other indices, market regimes, and time spans is still needed in the future work.

Despite these limitations, the empirical findings remain informative. The proposed model improves forecasting performance under a unified forward-looking protocol and shows stable advantages across multiple error measures and forecast horizons. In addition, the simple gross back testing analysis suggests that the model can provide useful stock-ranking information under a basic one-day-ahead top-10 long-only rule, although this result should be interpreted only as a preliminary gross diagnostic rather than as evidence of a fully optimized trading strategy. Overall, the evidence from the main experiments, ablation study, sensitivity analyses, multi-horizon DM tests, and simple gross back testing analysis supports a consistent conclusion: the superior performance of RST-HGA-GRU comes primarily from recurrent spatiotemporal fusion, while hypergraph attention provides an additional advantage through more effective modeling of higher-order stock dependence.

## 5. Conclusions and Future Work

This study proposes RST-HGA-GRU for stock price forecasting under a unified chronological setting. The model integrates hypergraph-based relational aggregation into the GRU state transition, enabling temporal dynamics and higher-order stock dependence to interact within the same recurrent update.

Experimental results show that RST-HGA-GRU achieves the best overall forecasting performance across multiple metrics and prediction horizons from 1 to 6 days. The ablation, sensitivity, and multi-horizon DM test results further indicate that both recurrent in-cell spatiotemporal fusion and tensor-decomposition-based hypergraph construction contribute to the final performance, while the simple gross back testing diagnostic provides preliminary evidence that the model also contains useful stock-ranking information under a basic one-day-ahead top-10 long-only rule.

Several limitations remain. The hypergraph construction still depends on feature design and decomposition settings, the experiments are restricted to a stable subset of CSI 300 constituent stocks, and broader validation across indices, market regimes, and time spans is still needed. In addition, the current back testing analysis does not incorporate transaction costs, turnover control, or portfolio risk constraints. Future work will extend the framework to broader market settings and examine more realistic transaction-cost-aware evaluation scenarios.

## Figures and Tables

**Table 1:** Stock symbols included in the dataset.

CSI 300	000001	000002	000063	000069	000100	000157	000338	000425
	000538	000568	000625	000651	000725	000768	000776	000858
	000876	000895	000963	002007	002142	002202	002230	002236
	002241	002304	002415	002594	600000	600009	600010	600015
	600016	600018	600019	600028	600029	600030	600031	600036
	600048	600050	600085	600104	600111	600115	600196	600276
	600309	600332	600362	600406	600519	600547	600585	600588
	600660	600690	600741	600795	600837	600886	600887	600893
	600900	600999	601006	601009	601088	601111	601166	601169
	601186	601288	601318	601328	601336	601377	601390	601398
	601600	601601	601618	601628	601633	601668	601669	601688
	601766	601800	601818	601857	601888	601899	601901	601939
	601988	601989	601998	603993				

**Table 2:** Hyperparameter settings of the proposed RST-HGA-GRU model.

Module	Hyperparameter	Candidate Values	Selected Value
HGA	in_channels	[16, 32, 64, 128, 256]	256
	hid_dim	[16, 32, 64, 128, 256]	32
	out_channels	[16, 32, 64, 128, 256]	256
	drop_rate	[0.2, 0.4, 0.6, 0.8]	0.4
	atten_neg_slope	0.5	0.5
	num_heads	4	4
GRU	input_size	1	1
	hidden_size	[16, 32, 64, 128, 256]	256
	out_size	1	1
	gru_layers	1	1
Others	optimizer	Adam	Adam
	epoch	500	500
	input_len	[1, 2, 3, 4, 5, 6, 7, 8]	6
	pre_len	[1, 2, 3, 4, 5, 6]	1
	batch_size	[64, 128, 256]	256
	learning_rate	[0.1, 0.01, 0.001]	0.001
	Weight_decay	[0.0001, 0.001, 0.01]	0.0001

**Table 3:** Optuna search spaces and selected hyperparameter settings for the tandem spatiotemporal baselines.

Hyperparameter	Search Space	ST-GCN-GRU	ST-GAT-GRU	ST-HGA-GRU
hid_dim	[16, 32, 64, 128, 256]	256	16	32
hidden_size	[16, 32, 64, 128, 256, 512]	512	128	512
gru_layers	[1, 2, 3]	3	3	1
drop_rate	[0.1, 0.2, 0.3, 0.4, 0.5, 0.6]	0.4	0.1	0.1
num_heads	[1, 2, 4, 8]	-	4	1
batch_size	[16, 128, 256]	64	64	64
learning_rate	[10^−4^, 5 × 10^−4^, 10^−3^, 5 × 10^−3^, 10^−2^]	5 × 10^−4^	5 × 10^−3^	10^−3^
Weight_decay	[0, 10^−5^, 10^−4^, 10^−3^]	10^−3^	10^−5^	0

Notes: hid_dim denotes the hidden dimension of the spatial aggregation module, whereas hidden_size denotes the hidden dimension of the recurrent unit. The hyperparameter num_heads applies only to attention-based baselines.

**Table 4:** Performance comparison between models.

Time	Model	*RMSE*	*MAE*	*MAPE*	*Acc*	*R* ^2^	*var*
1 day	ARIMA	0.0219	0.0143	0.0390	0.9619	0.9500	0.9501
	RFR	0.0965	0.0301	0.0590	0.8325	0.8598	0.9051
	SVR	0.0333	0.0264	0.1717	0.9422	0.7542	0.9573
	LSTM	0.0223	0.0133	0.0378	0.9614	0.9599	0.9661
	GRU	0.0231	0.0157	0.0443	0.9599	0.9418	0.9468
	ST-GCN-GRU	0.2471	0.1862	0.9564	0.5714	−7.3402	−0.2791
	ST-GAT-GRU	0.2238	0.1697	0.8061	0.6116	−5.9969	−0.3903
	ST-HGA-GRU	0.2144	0.1603	0.6461	0.6280	−4.8421	−0.1197
	RST-GCN-GRU	0.0171	0.0114	0.0296	0.9703	0.9700	0.9729
	RST-GAT-GRU	0.0163	0.0106	0.0310	0.9717	0.9727	0.9737
	RST-HGA-GRU	**0.0159**	**0.0102**	**0.0273**	**0.9724**	**0.9739**	**0.9741**
2 days	ARIMA	0.0261	0.0170	0.0470	0.9547	0.9274	0.9276
	RFR	0.1010	0.0337	0.0675	0.8248	0.8317	0.8826
	SVR	0.0349	0.0273	0.1752	0.9395	0.7545	0.9387
	LSTM	0.0262	0.0168	0.0455	0.9546	0.9405	0.9407
	GRU	0.0273	0.0179	0.0539	0.9527	0.9193	0.9201
	ST-GCN-GRU	0.2474	0.1860	0.9659	0.5707	−7.3845	−0.2783
	ST-GAT-GRU	0.2248	0.1722	0.8658	0.6100	−6.7956	−0.3314
	ST-HGA-GRU	0.2172	0.1650	0.6965	0.6230	−5.8846	−0.1134
	RST-GCN-GRU	0.0236	0.0156	0.0623	0.9591	0.9423	0.9555
	RST-GAT-GRU	0.0228	0.0149	0.0497	0.9605	0.9498	0.9569
	RST-HGA-GRU	**0.0198**	**0.0125**	**0.0325**	**0.9656**	**0.9604**	**0.9609**
3 days	ARIMA	0.0292	0.0190	0.0527	0.9493	0.9080	0.9084
	RFR	0.1047	0.0367	0.0747	0.8183	0.8035	0.8598
	SVR	0.0364	0.0283	0.1786	0.9368	0.7450	0.9271
	LSTM	0.0289	0.0178	0.0491	0.9496	0.9257	0.9259
	GRU	0.0306	0.0201	0.0589	0.9469	0.8943	0.9016
	ST-GCN-GRU	0.2477	0.1861	0.9689	0.5701	−7.3700	−0.2780
	ST-GAT-GRU	0.2256	0.1720	0.8436	0.6086	−6.5562	−0.3421
	ST-HGA-GRU	0.2144	0.1601	0.6160	0.6278	−4.5614	−0.2225
	RST-GCN-GRU	0.0330	0.0177	0.0444	0.9427	0.9392	0.9436
	RST-GAT-GRU	0.0266	0.0173	0.0499	0.9539	0.9314	0.9431
	RST-HGA-GRU	**0.0226**	**0.0144**	**0.0388**	**0.9607**	**0.9463**	**0.9471**

Notes: Bold indicates the optimal value. The same applies to the following highlighted entries.

**Table 5:** Repeated-run results under three random seeds after Optuna-based tuning.

Time	Model	*RMSE*	*MAE*	*MAPE*	*Acc*	*R* ^2^	*var*
1 day	ST-GCN-GRU	0.2065 ± 0.0022	0.1584 ± 0.0011	0.6650 ± 0.0185	0.6417 ± 0.0038	−5.5715 ± 0.7785	0.0541 ± 0.1158
	ST-GAT-GRU	0.1844 ± 0.0027	0.1312 ± 0.0012	0.4846 ± 0.0122	0.6801 ± 0.0047	−4.1189 ± 0.1340	−0.7481 ± 0.1118
	ST-HGA-GRU	0.2363 ± 0.0004	0.1799 ± 0.0002	0.9316 ± 0.0031	0.5901 ± 0.0007	−7.2512 ± 0.0366	−0.1159 ± 0.0250
2 days	ST-GCN-GRU	0.2086 ± 0.0025	0.1583 ± 0.0004	0.6603 ± 0.0251	0.6380 ± 0.0043	−5.3845 ± 0.3454	0.0393 ± 0.0742
	ST-GAT-GRU	0.1880 ± 0.0047	0.1346 ± 0.0045	0.5758 ± 0.0960	0.6737 ± 0.0082	−3.7758 ± 0.4228	−0.6063 ± 0.1441
	ST-HGA-GRU	0.2362 ± 0.0004	0.1790 ± 0.0011	0.9243 ± 0.0040	0.5902 ± 0.0008	−6.8609 ± 0.3349	−0.0843 ± 0.0300
3 days	ST-GCN-GRU	0.2078 ± 0.0031	0.1588 ± 0.0003	0.6701 ± 0.0179	0.6393 ± 0.0054	−5.6948 ± 0.5053	0.0360 ± 0.0183
	ST-GAT-GRU	0.1876 ± 0.0005	0.1326 ± 0.0007	0.4809 ± 0.0197	0.6744 ± 0.0008	−3.7506 ± 0.0804	−0.7574 ± 0.2929
	ST-HGA-GRU	0.2368 ± 0.0002	0.1800 ± 0.0002	0.9278 ± 0.0011	0.5891 ± 0.0004	−7.1362 ± 0.0924	−0.1061 ± 0.0365
4 days	ST-GCN-GRU	0.2067 ± 0.0009	0.1581 ± 0.0011	0.6678 ± 0.0098	0.6412 ± 0.0016	−5.5594 ± 0.5489	0.0070 ± 0.0454
	ST-GAT-GRU	0.1891 ± 0.0048	0.1358 ± 0.0043	0.5240 ± 0.0534	0.6718 ± 0.0084	−3.6600 ± 0.0603	−0.4456 ± 0.2542
	ST-HGA-GRU	0.2362 ± 0.0008	0.1791 ± 0.0013	0.9315 ± 0.0072	0.5900 ± 0.0013	−6.9799 ± 0.2770	−0.1009 ± 0.0221
5 days	ST-GCN-GRU	0.2076 ± 0.0005	0.1584 ± 0.0012	0.6612 ± 0.0056	0.6396 ± 0.0008	−5.5971 ± 0.4289	0.0204 ± 0.0786
	ST-GAT-GRU	0.1910 ± 0.0060	0.1347 ± 0.0040	0.5857 ± 0.0724	0.6684 ± 0.0103	−3.9835 ± 0.1530	−0.8797 ± 0.1812
	ST-HGA-GRU	0.2372 ± 0.0001	0.1803 ± 0.0001	0.9315 ± 0.0050	0.5882 ± 0.0002	−7.1403 ± 0.0587	−0.0964 ± 0.0251
6 days	ST-GCN-GRU	0.2080 ± 0.0004	0.1599 ± 0.0006	0.6915 ± 0.0371	0.6388 ± 0.0007	−5.9590 ± 0.0988	0.0127 ± 0.0847
	ST-GAT-GRU	0.1854 ± 0.0064	0.1306 ± 0.0054	0.5013 ± 0.0321	0.6781 ± 0.0111	−3.8505 ± 0.8100	−0.8090 ± 0.4493
	ST-HGA-GRU	0.2374 ± 0.0002	0.1804 ± 0.0002	0.9342 ± 0.0036	0.5878 ± 0.0003	−7.1676 ± 0.0934	−0.1016 ± 0.0337

**Table 6:** Performance comparison between models of ablation experiment.

Time	Model	*RMSE*	*MAE*	*MAPE*	*Acc*	*R* ^2^	*var*
1 day	ST-GCN-GRU	0.2471	0.1862	0.9564	0.5714	−7.3402	−0.2791
	ST-GAT-GRU	0.2238	0.1697	0.8061	0.6116	−5.9969	−0.3903
	ST-HGA-GRU	0.2144	0.1603	0.6461	0.6280	−4.8421	−0.1197
	RST-GCN-GRU	0.0171	0.0114	0.0296	0.9703	0.9700	0.9729
	RST-GAT-GRU	0.0163	0.0106	0.0310	0.9717	0.9727	0.9737
	RST-HGA-GRU (w/o TD)	0.0170	0.0121	0.0448	0.9706	0.9646	0.9738
	RST-HGA-GRU (Corr-HG)	0.0176	0.0111	0.0303	0.9694	0.9723	0.9736
	RST-HGA-GRU	**0.0159**	**0.0102**	**0.0273**	**0.9724**	**0.9739**	**0.9741**
2 days	ST-GCN-GRU	0.2474	0.1860	0.9659	0.5707	−7.3845	−0.2783
	ST-GAT-GRU	0.2248	0.1722	0.8658	0.6100	−6.7956	−0.3314
	ST-HGA-GRU	0.2172	0.1650	0.6965	0.6230	−5.8846	−0.1134
	RST-GCN-GRU	0.0236	0.0156	0.0623	0.9591	0.9423	0.9555
	RST-GAT-GRU	0.0228	0.0149	0.0497	0.9605	0.9498	0.9569
	RST-HGA-GRU (w/o TD)	0.0208	0.0148	0.0539	0.9639	0.9465	0.9606
	RST-HGA-GRU (Corr-HG)	0.0236	0.0146	0.0429	0.9590	0.9549	0.9597
	RST-HGA-GRU	**0.0198**	**0.0125**	**0.0325**	**0.9656**	**0.9604**	**0.9609**
3 days	ST-GCN-GRU	0.2477	0.1861	0.9689	0.5701	−7.3700	−0.2780
	ST-GAT-GRU	0.2256	0.1720	0.8436	0.6086	−6.5562	−0.3421
	ST-HGA-GRU	0.2144	0.1601	0.6160	0.6278	−4.5614	−0.2225
	RST-GCN-GRU	0.0330	0.0177	0.0444	0.9427	0.9392	0.9436
	RST-GAT-GRU	0.0266	0.0173	0.0499	0.9539	0.9314	0.9431
	RST-HGA-GRU (w/o TD)	0.0245	0.0171	0.0593	0.9575	0.9307	0.9466
	RST-HGA-GRU (Corr-HG)	0.0253	0.0160	0.0427	0.9560	0.9415	0.9457
	RST-HGA-GRU	**0.0226**	**0.0144**	**0.0388**	**0.9607**	**0.9463**	**0.9471**
4 days	ST-GCN-GRU	0.2190	0.1682	0.7337	0.6198	−6.6892	−0.1632
	ST-GAT-GRU	0.2256	0.1716	0.8346	0.6084	−6.3034	−0.3716
	ST-HGA-GRU	0.2479	0.1861	0.9684	0.5698	−7.2970	−0.2809
	RST-GCN-GRU	0.0302	0.0180	0.0525	0.9477	0.9263	0.9291
	RST-GAT-GRU	0.0281	0.0176	0.0502	0.9513	0.9285	0.9329
	RST-HGA-GRU (w/o TD)	0.0278	0.0194	0.0703	0.9518	0.9099	0.9325
	RST-HGA-GRU (Corr-HG)	0.0276	0.0173	0.0502	0.9521	0.9263	0.9306
	RST-HGA-GRU	**0.0257**	**0.0169**	**0.0511**	**0.9554**	**0.9283**	**0.9335**
5 days	ST-GCN-GRU	0.2193	0.1678	0.7269	0.6194	−6.4766	−0.1606
	ST-GAT-GRU	0.2256	0.1725	0.8545	0.6084	−6.7915	−0.3142
	ST-HGA-GRU	0.2478	0.1865	0.9764	0.5698	−7.5644	−0.2545
	RST-GCN-GRU	0.0336	0.0205	0.0568	0.9416	0.9077	0.9164
	RST-GAT-GRU	0.0308	0.0195	0.0518	0.9465	0.9080	0.9097
	RST-HGA-GRU (w/o TD)	0.0312	0.0216	0.0800	0.9458	0.8890	0.9180
	RST-HGA-GRU (Corr-HG)	0.0340	0.0212	0.0564	0.9410	0.8980	0.9136
	RST-HGA-GRU	**0.0288**	**0.0185**	**0.0504**	**0.9500**	**0.9134**	**0.9179**
6 days	ST-GCN-GRU	0.2148	0.1611	0.6618	0.6270	−5.0004	−0.1938
	ST-GAT-GRU	0.2261	0.1723	0.8460	0.6074	−6.5575	−0.3456
	ST-HGA-GRU	0.2480	0.1855	0.9476	0.5693	−7.0059	−0.2611
	RST-GCN-GRU	0.0344	0.0220	0.0688	0.9403	0.8902	0.9044
	RST-GAT-GRU	0.0345	**0.0211**	0.0616	0.9402	**0.8978**	0.9013
	RST-HGA-GRU (w/o TD)	0.0353	0.0239	0.0881	0.9387	0.8694	**0.9032**
	RST-HGA-GRU (Corr-HG)	0.0336	0.0240	0.0751	0.9416	0.8549	0.8938
	RST-HGA-GRU	**0.0322**	0.0212	**0.0601**	**0.9440**	0.8908	0.9022

## Data Availability

The data of the study is available at https://github.com/linfengqian/stock_data-csi-300- (accessed on 5 February 2026).
